# Identification of the elementary structural units of the DNA damage response

**DOI:** 10.1038/ncomms15760

**Published:** 2017-06-12

**Authors:** Francesco Natale, Alexander Rapp, Wei Yu, Andreas Maiser, Hartmann Harz, Annina Scholl, Stephan Grulich, Tobias Anton, David Hörl, Wei Chen, Marco Durante, Gisela Taucher-Scholz, Heinrich Leonhardt, M. Cristina Cardoso

**Affiliations:** 1 Department of Biology, Technische Universität Darmstadt, 64287 Darmstadt, Germany; 2 Department of Biology II, Center for Integrated Protein Science Munich (CIPSM), LMU Munich, 82152 Planegg-Martinsried, Germany; 3 Max Delbrück Center for Molecular Medicine, 13125 Berlin, Germany; 4 Department of Biophysics, GSI Helmholtzzentrum für Schwerionenforschung, 64291 Darmstadt, Germany

## Abstract

Histone H2AX phosphorylation is an early signalling event triggered by DNA double-strand breaks (DSBs). To elucidate the elementary units of phospho-H2AX-labelled chromatin, we integrate super-resolution microscopy of phospho-H2AX during DNA repair in human cells with genome-wide sequencing analyses. Here we identify phospho-H2AX chromatin domains in the nanometre range with median length of ∼75 kb. Correlation analysis with over 60 genomic features shows a time-dependent euchromatin-to-heterochromatin repair trend. After X-ray or CRISPR-Cas9-mediated DSBs, phospho-H2AX-labelled heterochromatin exhibits DNA decondensation while retaining heterochromatic histone marks, indicating that chromatin structural and molecular determinants are uncoupled during repair. The phospho-H2AX nano-domains arrange into higher-order clustered structures of discontinuously phosphorylated chromatin, flanked by CTCF. CTCF knockdown impairs spreading of the phosphorylation throughout the 3D-looped nano-domains. Co-staining of phospho-H2AX with phospho-Ku70 and TUNEL reveals that clusters rather than nano-foci represent single DSBs. Hence, each chromatin loop is a nano-focus, whose clusters correspond to previously known phospho-H2AX foci.

## Abstract (web summary)

Phosphorylated histone H2AX is an early signalling event of DNA double-strand breaks. Here the authors use super-resolution microscopy and ChIP-seq and identify ‘nano-domains' – chromatin loops decorated by γH2AX and flanked by CTCF.

DNA double-strand breaks (DSBs) are the most harmful lesions induced by either endogenous (for example, replication) or exogenous (for example, ionizing radiation-IR) genotoxic stress, which may lead to chromosomal aberrations and tumorigenesis if not correctly repaired. To deal with DSBs, cells activate a rapid and hierarchically coordinated signalling cascade known as DNA damage response (DDR), leading to cell cycle arrest and allowing the DNA repair machinery to exert its function. One of the earliest events of DDR is the phosphatidylinositol-3-kinase-like-dependent phosphorylation of serine 139 of histone H2AX (γH2AX)^1^, a histone H2A variant whose role at the interface of DNA repair, chromatin structure regulation and cell cycle checkpoint activation^2^ is yet to be fully elucidated.

Detection of γH2AX has become the most widely used method for quantification of DSBs and their repair kinetics. Activated DDR, as scored by quantification of nuclear γH2AX focal structures, has been extensively described in both precancerous and cancer cells^3^,^4^. The majority of these studies were performed by conventional microscopy techniques, including confocal microscopy, and the structures resolved were in the micrometre or sub-micrometre range, with a predicted DNA content in the megabase-pair (Mbp) range. Indeed, γH2AX is proposed to spread up to several Mbps from the original lesion site, in higher eukaryotes^5^. The distribution of such histone modification is neither symmetrical around DSB sites nor uniform on chromatin, as assessed by chromatin immunoprecipitation (ChIP) studies conducted in mammals^6^,^7^,^8^ and yeast^9^,^10^. Such uneven spreading may be accounted for by gene transcription^11^, or cohesin complex binding^12^, which antagonize γH2AX formation along the chromosomes.

An increasing body of evidence underlines the crucial role of genome topology and chromatin spatial organization in the regulation of biological processes^13^. Recent chromosome conformation capture studies have revealed the complexity of genome architecture, with large compartments in the Mbp range conserved across cell lineages and species^14^,^15^, as well as smaller contact domains with a variable size in the range of a few hundreds of kilobase pairs (kb)^15^. This spatial organization can be dynamic and underlines cell-type-specific networks, possibly driving the expression of specific sets of genes^16^ or organizing the replication process^17^.

Nonetheless, the three-dimensional (3D) arrangement of γH2AX-decorated chromatin in the nuclear volume and its dynamic evolution during the DDR remains elusive. Here we investigate the DDR over time at nanometre resolution by employing super-resolution microscopy techniques on human cells exposed to X-ray radiation. By overcoming the optical diffraction limit, structured illumination microscopy (3D-SIM)^18^ and stimulated-emission-depletion (STED)^19^ fluorescence microscopy present high prospecting capacity, thus allowing us to dissect complex structures of γH2AX-decorated chromatin at nanometre resolution (∼100 nm). Furthermore, the integration of the microscopy results with CRISPR-Cas-targeted DNA damage, RNAi of the key structural factor CCCTC-binding factor (CTCF), γH2AX ChIP-Seq(uencing) profiles during DDR, and more than 60 genomic features reveal temporal, functional and structural insights into the elementary chromatin units read by the DNA DSB repair machinery.

## Results

### Cellular system and experimental strategy validation

For our study, we employed HeLa cells, an established human cell line whose (epi)genome is extensively annotated in the context of the ENCODE project (genome.ucsc.edu/ENCODE/). To test the DDR, we assessed the formation of γH2AX before and after exposure to IR. We investigated the early (0.5 h), mid (3 h) and late (24 h) stages of DDR, which, according to earlier reports^20^, represent 60–100%, 20–60% and less than 10% of the initial DSBs, respectively. Our confocal immunofluorescence analysis of γH2AX revealed that the show endogenous γH2AX signal. This is frequently observed in cancer cell lines and can be attributed to randomly produced DSBs at stalled and collapsed replication forks^21^,^22^. On exposure to IR, γH2AX followed the predicted repair kinetics, with nuclear γH2AX fluorescence intensity increasing, and then decreasing over time (Supplementary Fig. 1A). Similar kinetics was observed by western blot analysis (Supplementary Fig. 1B). Together, these methods revealed a four- to eightfold increase in γH2AX signal after IR. Overall, cells were able to activate a DDR and underwent cell cycle arrest, accumulating in S-phase (Supplementary Fig. 1C). No apoptosis was detected (Supplementary Fig. 1D), and 24 h post IR cells were viable, re-entered the cell cycle (Supplementary Fig. 1C) and proliferated, although at a lower rate compared with the mock-irradiated controls (Supplementary Fig. 1E).

**Figure 1:**
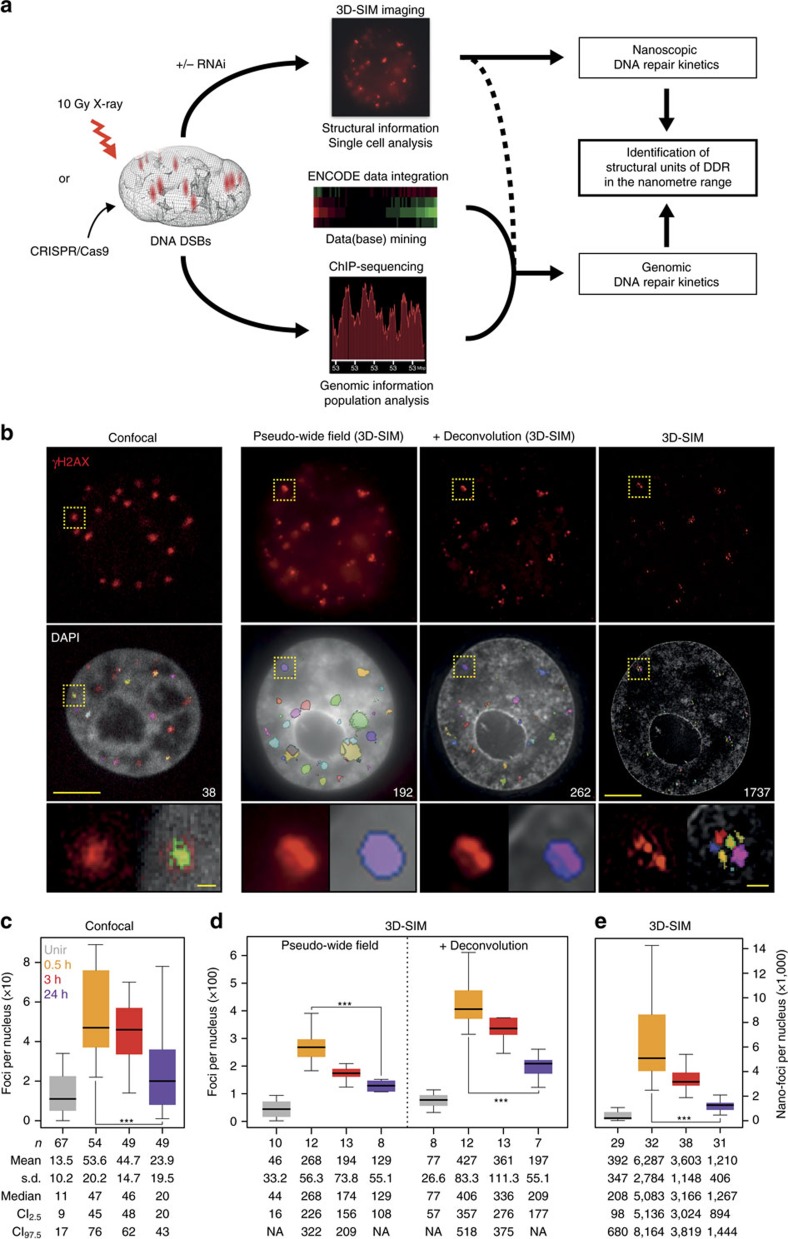
Characterization of γH2AX foci at different resolution levels. (**a**) Schematics of the experimental approach. (**b**) Mid-nuclear sections of confocal microscopy (*z*: 200 nm) and 3D-SIM (*z*: 125 nm) representative images of cells, 24 h post IR. Only for 3D-SIM, the same exemplary cell is shown as re-computed pseudo-wide-field image before or after deconvolution as well as the original 3D-SIM output. The total number of detected foci (highlighted in colours) in the whole nuclear volume is shown in the DAPI panels. The lower panels show magnified views of the yellow dashed frame. Scale bars, 5 μm and 500 nm for main micrographs and magnified regions, respectively. γH2AX foci number distributions before and during DDR, from confocal images (**c**), 3D-SIM re-computed pseudo-wide-field of identical cell nuclei, before or after deconvolution (**d**) and original 3D-SIM images (**e**). *n*: total number of imaged cells from three independent experiments. All boxes and whiskers represent 25–75 percentiles and three times the IQD. The mean number of foci and corresponding s.d., the median as well as the 95% confidence intervals (CI) for the median are shown below each box. NA: not applicable. For **c**–**e**: one-way ANOVA with Dunnett's correction; ***P<10^−3^.

To investigate γH2AX kinetics at high resolution, we recorded super-resolution image sets before and during DDR, and acquired γH2AX ChIP-Seq genome-wide data at matching time points (Fig. 1a). In all of our immuno-based approaches, we probed γH2AX-decorated chromatin with the same antibody, whose specificity was verified by slot blot analysis employing the γH2AX-immunizing peptide (Supplementary Fig. 1F). The reproducibility of the sequencing data was assessed and confirmed by comparing biological replicates (Supplementary Fig. 1G).

### Super-resolution microscopy of γH2AX kinetics during DDR

To first address the effect of improved optical resolution, we compared the number of γH2AX foci from cells imaged by conventional confocal and 3D-SIM microscopy, and analysed in addition the pseudo-wide-field images re-computed from the same 3D-SIM images, before and after deconvolution (Fig. 1b). A detailed analysis workflow is in the ‘Methods' section and summarized in Supplementary Fig. 1H. Compared with confocal images (Fig. 1c), we observed a fivefold increase in foci numbers in pseudo-wide-field images, with an additional twofold increase in deconvolved images (Fig. 1d). Despite employing IR doses that are challenging for conventional confocal microscopy (10 Gy X-ray), the enhanced optical resolution enabled us to resolve thousands of foci, increasing by about one order of magnitude the foci counts compared with the pseudo-wide-field, and about two orders of magnitude when comparing with confocal microscopy (Fig. 1e). Thus, it becomes obvious that a single focus identified by confocal microscopy can be further resolved by 3D-SIM into substructures (Fig. 1b, bottom panels, and Supplementary Fig. 2A), which we referred to as nano-foci. In addition, we controlled the imaging and reconstruction process of 3D-SIM by visual inspection of the reconstructed images in Fourier's space (Supplementary Fig. 2B). No reconstruction artifacts are visible as can be seen from the fast Fourier transformed images, which would contain regular stripe patterns otherwise.

**Figure 2:**
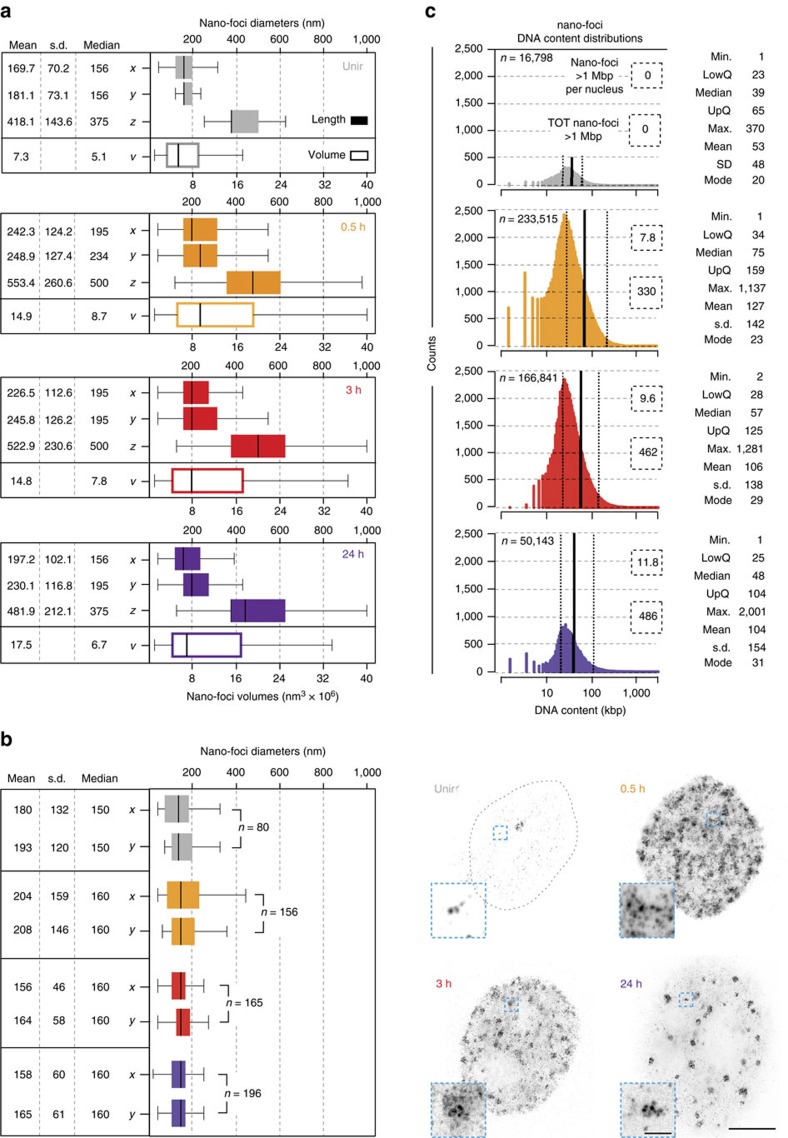
Metrics of γH2AX nano-foci dimensions and DNA content. (**a**) Quantification of nano-foci diameters in the three dimensions (filled boxes, top) during DDR. From these three dimensions, the volumes were calculated (empty boxes, bottom). The difference between lateral and axial measurements is due to the lower resolution in the axial direction. Figures in nm or nm^3^ × 10^6^ are shown. (**b**) STED microscopy of γH2AX immunofluorescence. (left) Quantification of lateral diameters of γH2AX nano-foci. Statistics and size scale are as in **a**. (right) Exemplary STED images of cells before and after IR are shown together with the magnified views of the light-blue boxes. Scale bars, 5 μm and 500 nm for main micrographs and magnified regions, respectively. (**c**) DNA content distributions of γH2AX nano-foci before and during DDR. Only in IR-exposed cells, we found nano-foci larger than 1 Mbp (dashed boxes), and their frequency never exceeded 1% (0.14%, 0.28%, 0.95% for 0.5 h, 3 h and 24 h, respectively). Kruskal–Wallis *χ*^2^=18,503, df=3, *P*<2.2 × 10^−16^. Statistics (in kb) are shown next to each distribution. All boxes and whiskers are as in Fig. 1. *n*: total number of measured nano-foci from all imaged cells in two independent experiments, for 3D-SIM (**a**,**c**) or STED (**b**).

Coherently, we observed a two- to fourfold decrease in the diameters of the segmented objects, when comparing 3D-SIM images with re-computed pseudo-wide-field images, with or without deconvolution, respectively (Supplementary Fig. 3A). Notably, in the 3D-SIM images, the nano-foci diameters were constant during the DDR (median lateral diameter: ∼200 nm; Fig. 2a), indicating that we detected the smallest substructures of γH2AX-decorated chromatin at the limit defined by the foci segmentation process (eight voxels). To gauge the actual size of γH2AX nano-foci, we recorded γH2AX immunofluorescence images by STED microscopy. Compared with our 3D-SIM set-up, STED provided a twofold increase in optical resolution^18^. Yet, the measured lateral diameters (Fig. 2b) were statistically undistinguishable from those recorded by 3D-SIM under sham-irradiation conditions (unpaired two-tailed *t*-test: *P*>0.05). Upon irradiation, the mean lateral diameters imaged by STED were only ∼20% smaller than those we measured by 3D-SIM imaging (unpaired two-tailed *t*-test: *P*<10^−3^). These results validate our 3D-SIM measurements and indicate that γH2AX nano-foci are the chromatin elementary units of the cellular response to DSBs.

Next, to estimate the DNA content of nano-foci, we related the integrated 4,6-diamidino-2-phenylindole (DAPI) intensity of each γH2AX nano-focus to the total DNA content represented by the integrated whole nuclear DAPI intensity (Supplementary Fig. 3B). The resulting DNA fractions were first corrected for the total HeLa genome size (determined by spectral karyotyping, Supplementary Fig. 3C), and then further corrected for the cell cycle phase of each given cell (Supplementary Fig. 3D). Finally, values smaller than the 0.5th and bigger than 99.5th percentile were discarded to avoid artifactual biases. The resulting distributions are shown in Fig. 2c. Before exposure to IR, the interquartile distance (IQD) of the nano-foci DNA content was ∼23–65 kb. On IR (0.5 h)—after γH2AX spreading—it increased to ∼34–159 kb, with a median length of 75 kb (Fig. 2c and Supplementary Tables 1 and 2).

To provide another line of evidence supporting our 3D-SIM metrics, we produced γH2AX ChIP-Seq profiles under the same experimental conditions employed for the microscopic analysis. Next, we integrated the genomic data with the super-resolution microscopy data to establish a novel combined approach (described in detail in the Methods and Supplementary Fig. 4) and, thus, provide estimates of the γH2AX-decorated chromatin domain size. Overall, the resulting γH2AX genomic domains' size was in good agreement with that of 3D-SIM γH2AX nano-foci, although the former were ∼30% smaller (IQD: 10–110 kb at 0.5 h). Because our approach only takes into account the *in cis* contribution to the size of the genomic domains, the difference between the latter and those measured by 3D-SIM can be attributed to inter-chromosomal contribution^23^.

### The DDR uncouples histone modifications and DNA compaction

**Figure 3:**
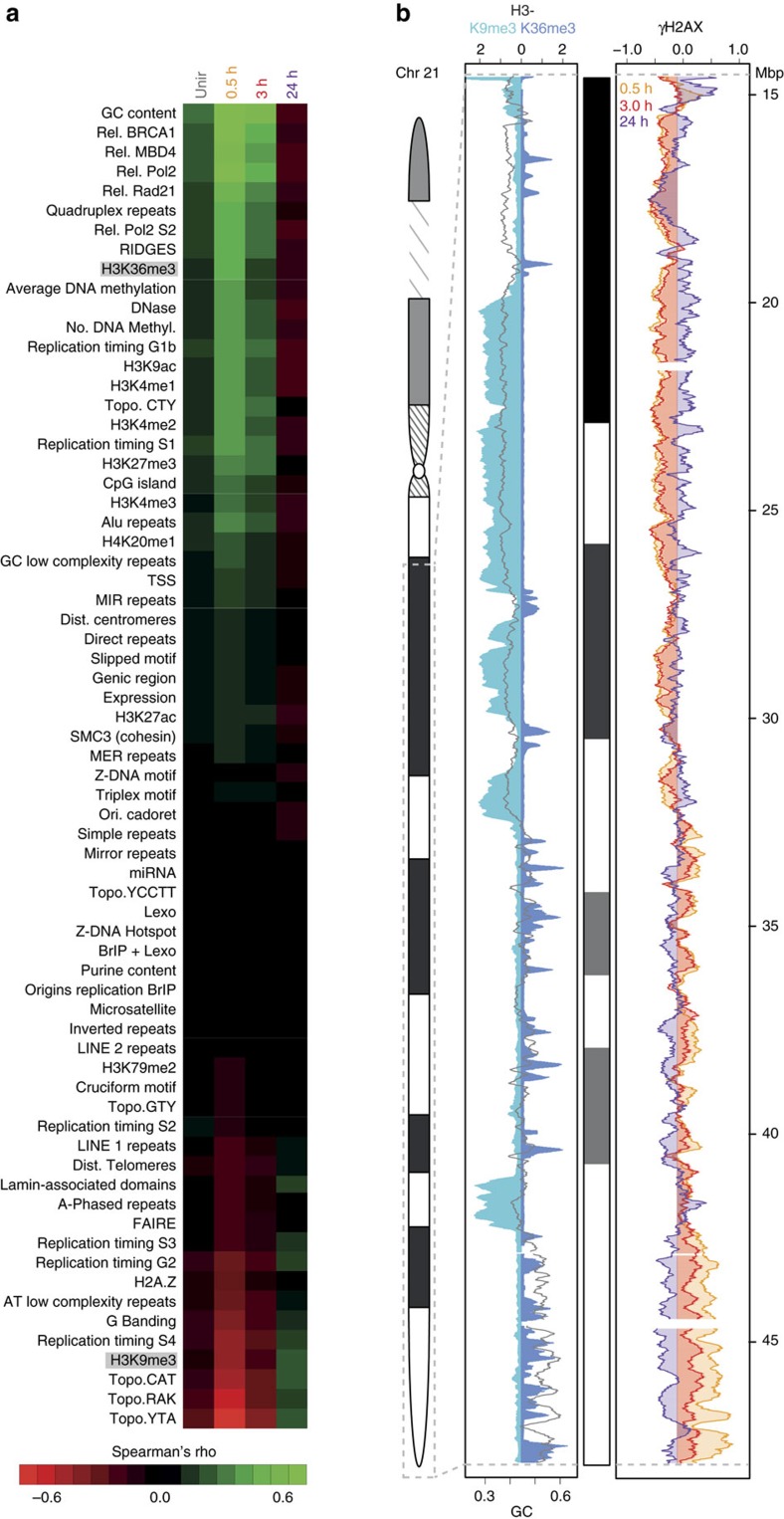
Temporal correlation of γH2AX ChIP-Seq signal and genomic features. (**a**) Genome-wide correlation between ChIP-Seq γH2AX profiles and genomic features, before and during DDR. Spearman's *ρ* correlation coefficient is calculated between 10 kb-binned γH2AX profiles and the genomic features (Supplementary Table 3), and colour-coded from red (anti-correlation) to green (correlation). All genomic features are ordered decreasingly, according to the highest correlation value (γH2AX and GC, 0.5 h: 0.81). For all correlations: *P*<<2.2 × 10^−16^. (**b**) Exemplary ChIP-Seq γH2AX profile on chromosome 21. (left) H3K9me3, H3K36me3 and GC content (grey line); (right) γH2AX levels during DDR.

To characterize the (epi)genetic composition of γH2AX-decorated chromatin during DDR, we related the ChIP-Seq γH2AX profiles to multiple genomic features, (Supplementary Table 3). First, we computed the density of such genomic features as well as the abundance of γH2AX in 10 kb genomic intervals. Next, we calculated the genome-wide Spearman's *ρ* correlation coefficient of each feature with γH2AX profiles before and during the DDR (Fig. 3a). The outcome of the analysis showed a strong correlation at early time post IR between γH2AX and euchromatic features such as GC content (Supplementary Fig. 5A; maximum Spearman's *ρ*: 0.81, *P*<2.2 × 10^−16^), DNase hypersensitivity sites, Regions of IncreaseD Gene Expression (RIDGEs), early replication timing and histone modifications associated with transcriptionally active chromatin state (for example, H3K36me3, H3K4me1/2/3 and H3K9ac). Heterochromatic features, such as AT content (Topo.CAT-YTA-RAK motif), lamin-binding sites, late replication timing, intensity of Giemsa shades and H3K9me3, were negatively correlated to γH2AX, instead. Notably, this trend was inverted at later times, with heterochromatic features correlating to residual γH2AX levels. An exemplary γH2AX profile on chromosome 21 is shown in Fig. 3b. Quantification of γH2AX levels, before and during DDR, in (anti-)RIDGEs, Giemsa shades as well as in H3K36me3- and H3K9me3-decorated chromatin domains is shown in Supplementary Fig. 5B–D and Supplementary Table 4.

**Figure 4:**
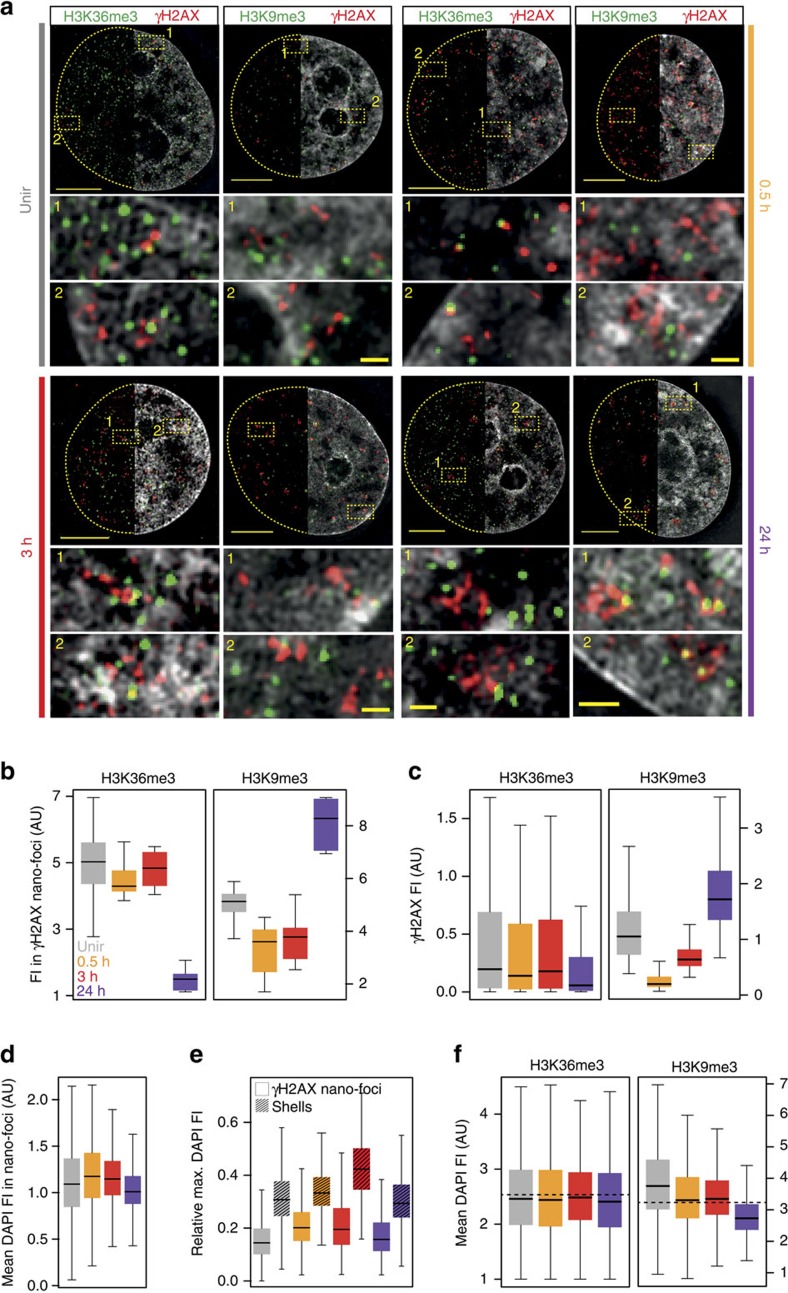
3D-SIM chromatin composition analysis of γH2AX nano-foci before and during DDR. (**a**) Exemplary 3D-SIM images of γH2AX (red) and H3K9me3/H3K36me3 (green) co-immunostaining before and after IR. Top panels: mid-nuclear sections showing γH2AX and histone marks with (right half) or without (left half) DAPI counterstaining. The dashed lines depict the nuclear contour. Bottom panels: magnification of the yellow dashed boxes with corresponding reference number. Scale bars, 5 μm and 500 nm for main micrographs and magnified regions, respectively. (**b**) Quantification of the H3K36me3 and H3K9me3 fluorescence intensities measured in γH2AX nano-foci volumes. Kruskal–Wallis *χ*^2^=19.875, df=3, *P*=1.802 × 10^−4^ and Kruskal–Wallis *χ*^2^=24,451, df=3, *P*=2.011 × 10^−5^. (**c**) Quantification of the γH2AX fluorescence intensity in H3K36me3- (Kruskal–Wallis *χ*^2^=261,960, df=191,020, *P*<2.2 × 10^−16^) and H3K9me3- (Kruskal–Wallis *χ*^2^=246,300, df=232,750, *P*<2.2 × 10^−16^) decorated chromatin. (**d**) Mean DAPI intensity in γH2AX nano-foci. Kruskal–Wallis *χ*^2^=247,910, df=245,320, *P*=1.129 × 10^−4^. (**e**) Quantification of maximum DAPI intensity in the volume occupied by γH2AX nano-foci (regular boxes) and shells (pattern), relative to the maximum integrated nuclear intensity. Shells represent 3D hollow structures surrounding γH2AX nano-foci (Supplementary Fig. 5E and ‘Methods' section). Wilcoxon rank sum all <2.2 × 10^−16^. (**f**) Mean DAPI fluorescence intensity in H3K36me3- or H3K9me3-decorated chromatin. Kruskal–Wallis *χ*^2^=303,050, df=292,700, *P*<2.2 × 10^−16^ and Kruskal–Wallis *χ*^2^=25,500, df=25,002, *P*=0.01338. Dotted lines: mean DAPI intensity measured over the whole analysed nuclei. All boxes and whiskers are as in Fig. 1. AU: arbitrary units. Results are from two independent experiments.

To validate and extend these findings at the single-cell level, we recorded 3D-SIM images of γH2AX immunofluorescence combined with either H3K36me3 or H3K9me3 labelling (Fig. 4a). These two histone modifications recapitulate the results from Fig. 3a, with the former being mainly associated with actively transcribed genes^24^, while the latter is abundant in heterochromatic (for example, pericentromeric regions) and transcriptionally silent regions^25^. We segmented γH2AX nano-foci as previously described and, in addition, we measured the H3K36me3 or H3K9me3 fluorescence intensity in the volume occupied by γH2AX nano-foci. In the latter, H3K36me3 signal was high at early time points, but not at 24 h post IR, as opposed to H3K9me3 signal, which was low at early time points but higher 24 h post IR (Fig. 4b). We observed similar results when measuring γH2AX fluorescence intensity in the volume of H3K36me3- and H3K9me3-decorated chromatin (Fig. 4c). Together, these findings recapitulate our genomic results, indicating that γH2AX nano-foci are mainly associated to an active chromatin state during the early and mid-stages of DDR, whereas the residual phosphorylation signal is enriched in heterochromatin at later times.

**Figure 5:**
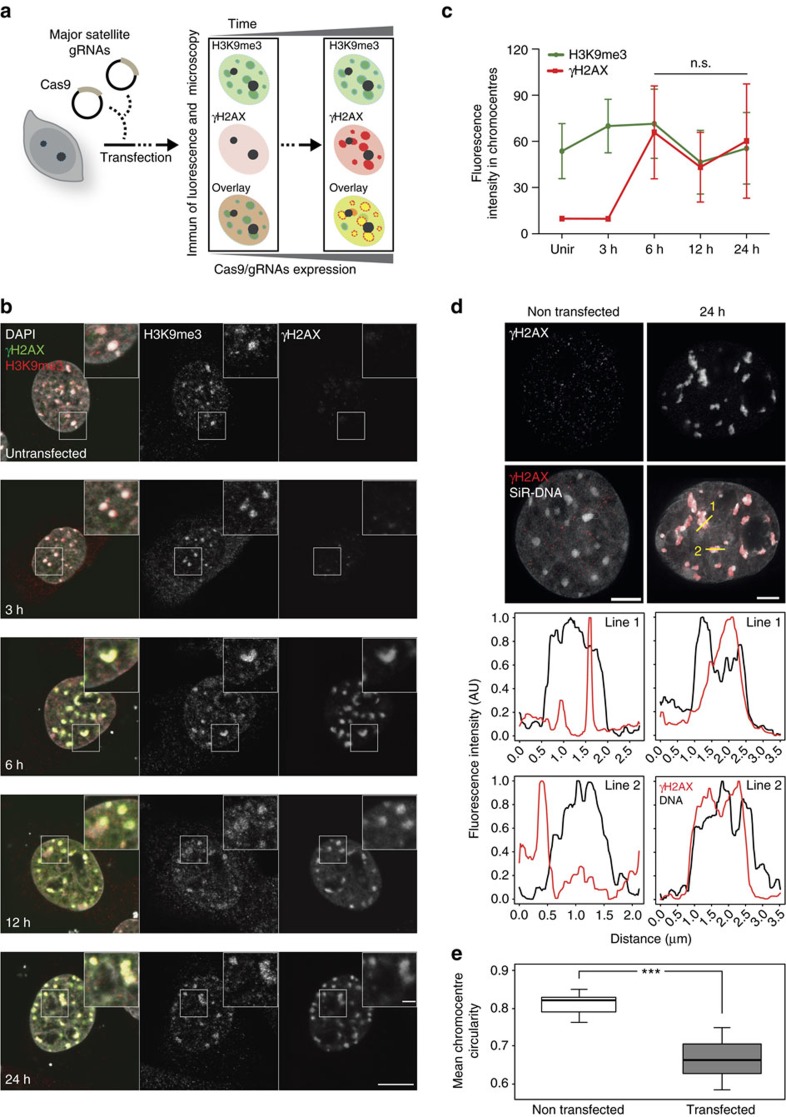
Analysis of γH2AX and H3K9me3 levels at heterochromatin-targeted CRISPR-Cas9-mediated DSBs. (**a**) Schematics of the CRISPR-Cas9-mediated DSBs induction at murine major satellites DNA. C2C12 cells were transfected with Cas9 and major satellites gRNAs plasmids and fixed after the indicated times. (**b**) Representative immunofluorescence images of γH2AX and H3K9me3 in C2C12 cells. Scale bars, 10 μm and 2 μm for micrograph and inset, respectively. (**c**) Quantification of γH2AX and H3K9me3 fluorescence intensity from DAPI-segmented chromocentres. Mean and s.d. from (**b**) are shown. *n*=5 cells (2–19 chromocentres), 5 cells (6–15 chromocentres), 5 cells (8–19 chromocentres), 5 cells (13–19 chromocentres) and 4 cells (9–19 chromocentres), for untransfected, 3 h, 6 h, 12 h and 24 h time points, respectively. See image analysis in the ‘Methods' section for details. (**d**) Representative STED immunofluorescence images of γH2AX and SiR-labelled DNA as indicated. Yellow lines: line profiles (shown below). For the latter, fluorescence intensities were normalized to the min–max range of values of each profile. Lines were smoothed by a 5-window running median. (**e**) Chromocentres decondensation after major satellite-targeted Cas9, assessed as mean chromocentre circularity in transfected (*n*=9) and untransfected (*n*=10) cells. For each cell, the circularity of chromocentres (>100 px^2^) within the nucleus was determined as described in the ‘Methods' section, yielding shape information for 165 (transfected cells) and 148 (untransfected cells) chromocentres. Statistics: Wilcoxon rank sum test (***P<10^−3^). Scale bar, 2 μm.

Based on these data, we expected an enrichment of γH2AX nano-foci in compact chromatin (that is, DAPI-dense structures) at later times. However, the mean DAPI content of γH2AX nano-foci remained unvaried over the time, and, if at all, was lower at 24 h (Fig. 4d). In fact, γH2AX nano-foci were located in close proximity to DAPI-dense structures, and the two seldom overlapped. To quantify this, we measured the maximum DAPI intensity in a 3D-region dilated by three voxels in all dimensions around each γH2AX nano-focus, which we referred to as ‘shell' (Supplementary Fig. 5E). Shells always presented higher DAPI signal than the nano-foci (Fig. 4e). This is in agreement with previous observations, whereby γH2AX-decorated chromatin was excluded from DAPI-dense structures following DSB induction^26^,^27^. These findings prompted us to investigate the condensation state of H3K9me3-decorated chromatin after DNA damage induction. On IR, we observed a progressive decrease of DAPI intensity in H3K9me3-decorated chromatin, up to 24 h (Fig. 4f). Such decrease was not observed in H3K36me3-decorated chromatin. Together, this implies that heterochromatic regions underwent DNA decondensation, although they retained their histone marks. To independently validate this finding, we investigated γH2AX and H3K9me3 levels before and after the induction of CRISPR-Cas9-mediated DNA DSBs targeted at heterochromatic murine major satellite repetitive DNA elements, in C2C12 cells (Fig. 5a). These genomic regions are predominantly found at H3K9me3-rich chromatin and are the most condensed chromatin domains in the mouse genome (chromocentres). As early as the ectopically expressed Cas9 was active (>3 h), γH2AX was visible at H3K9me3-decorated chromatin (chromocentres) (Fig. 5b). Quantification of the H3K9me3 and γH2AX fluorescence intensity in the segmented chromocentres revealed that both signals co-localized (Fig. 5c). Next, we analysed the condensation state of Cas9-targeted chromocentres by means of dual-colour STED microscopy and DNA density measurements. On Cas9-mediated DSBs induction, chromocentres were dramatically decondensed (Fig. 5d,e). Remarkably, they retained the γH2AX mark, which was more abundant where the DNA signal was diminished (Fig. 5d). This observation is in agreement with our 3D-SIM data, whereby the γH2AX nano-foci present a partially decondensed state, with diminished DNA levels relative to their surroundings (Fig. 4f).

Taken together, these findings show that γH2AX nano-foci are chromatin units over represented in transcriptionally active regions early on exposure to IR. During the late stage of DDR, they mark heterochromatic regions whose DNA is in a locally decondensed state while keeping the characterizing histone marks (for example, H3K9me3). We propose that by retaining their histone mark, the chromatin identity of such domains is preserved. This not only indicates that the actual chromatin compaction state can be uncoupled from the histone modifications of a given chromatin domain, but also it suggests a modality to reestablish the original chromatin state, once DNA repair is accomplished.

### γH2AX foci consist of spatially clustered γH2AX nano-foci

**Figure 6:**
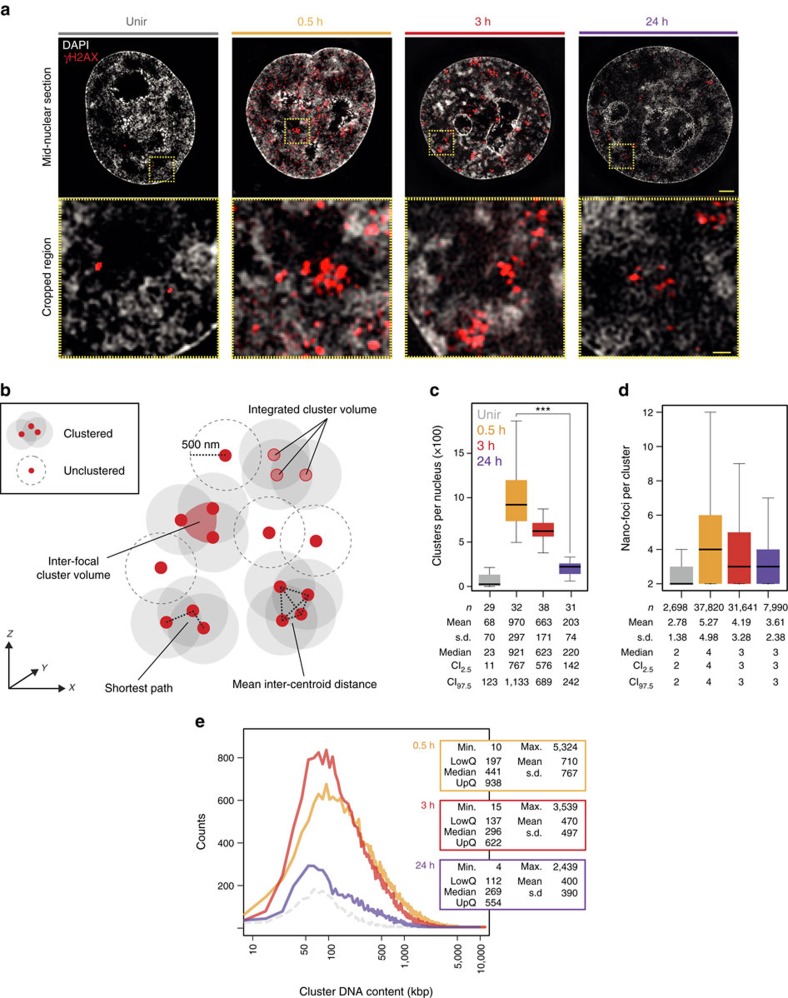
Analysis of γH2AX nano-foci spatial clustering. (**a**) Exemplary 3D-SIM images of γH2AX immunofluorescence before and during DDR. Shown are the mid-nuclear section with DAPI and γH2AX signals, and magnified view from the yellow frame. Scale bars, 2 μm and 400 nm for main micrographs and magnified regions, respectively. (**b**) Schematics of γH2AX 3D-clusters analysis. All centroids (red dots) within a sphere defined by a given cutoff radius (500 nm in further analysis) are included in a cluster. For all nano-foci belonging to each given cluster, the sum of the volume of single nano-foci (integrated cluster volume), the volume delimited by the centroids (inter-focal volume), the shortest path connecting all centroids as well as the mean distance between centroids (mean inter-centroid distance) are computed (Supplementary Fig. 6C–F). (**c**) γH2AX 3D-clusters per nucleus. One-way ANOVA with Dunnett's correction; ***P<10^−3^. (**d**) γH2AX nano-foci per 3D-clusters. Kruskal–Wallis *χ*^2^=1,926.3, df=3, *P*<2.2 × 10^−16^. (**e**) DNA content distributions of γH2AX 3D-clusters during DDR. The DNA content of each nano-focus belonging to a given cluster is summed. The dashed line depicts the distribution of γH2AX 3D-clusters before IR. Kruskal–Wallis *χ*^2^=5,964.1, df=3, *P*<2.2 × 10^−16^. Statistics are presented as in Fig. 2. All boxes and whiskers are as in Fig. 1. *n*: number of analysed cells (**c**) or 3D-clusters (**d**).

On exposure to IR, and as DDR progressed, γH2AX nano-foci were distributed throughout the nuclear volume, though they appeared to be spatially clustered (Fig. 6a and Supplementary Fig. 6A). To investigate such spatial clustering, we reconstructed the position of γH2AX nano-foci in the 3D nuclear space by collecting their 3D coordinates. Next, we measured the distances between the centroid of each nano-focus and all the other nano-foci in the nucleus. If the centroids of two objects were closer than a given cutoff distance, we assigned the corresponding nano-foci to the same cluster (Fig. 6b and Supplementary Fig. 1H and ‘Methods' section). Based on the median lateral nano-focus radius of ∼100 nm, we reasoned that two adjacent nano-foci would be spatially positioned so that their centroids would be at least 200 nm (2 × radius) away. Indeed, cutoff distances smaller than 300 nm resulted in poor clustering (Supplementary Fig. 6B). Similarly, distances bigger than 700 nm reduced the number of clusters at all time points, cancelling out differences over the time and, hence, impeding the analysis of the repair kinetics (Supplementary Fig. 6B). A cutoff distance of 500 nm (Fig. 6b) resulted in the highest number of clusters and a clear repair kinetics (Fig. 6c and Supplementary Table 5). Overall, the number of clusters was significantly higher than that of foci resolved by confocal microscopy, and comparable to the number of foci observed in pseudo-wide-field images (Fig. 1b,d). After IR, clusters were composed of a median number of four nano-foci (Fig. 6d), with the distributions remaining remarkably similar for all time points. This indicates that at times when the DSBs are repaired, the complete clusters, rather than single nano-foci, are removed en bloc. Coherently, clusters had an integrated median volume of about 0.05 μm^3^ (Supplementary Fig. 6C), which decreased at later times. The average inter-centroid distance measured between all nano-foci belonging to a given cluster, the shortest path connecting all the centroids in a given cluster, and the inter-focal volume delimited by the 3D coordinates of the centroids of each nano-focus belonging to a cluster showed similar kinetics (Fig. 6b and Supplementary Fig. 6D–F). In all cases, these parameters increased after IR and then decreased, indicating that the nano-foci in each cluster were progressively closer to one another as the DDR progressed. One possible explanation is an active chromatin structure change bringing the clustered nano-foci in close proximity and, thus, facilitating the repair process of complex lesions at later times. However, the possibility that the clusters repaired at later times might correspond to a subset of damaged chromatin fibres whose location was in close spatial proximity already at earlier times is equally possible.

Finally, based on the previous nano-foci DNA content estimates, we calculated the DNA content of clusters by summing the DNA content of all γH2AX nano-foci belonging to a given cluster (Supplementary Tables 1 and 2). After IR, we observed broad-size distributions, with IQDs of about 197–938, 137–622 and 112–554 kb for 0.5 h, 3 h and 24 h time points, respectively (Fig. 6e). Overall, the cluster DNA content is in the (sub-)Mbp range, being directly relevant to genome regulation processes, as reported by genomic^14^,^15^,^17^ or super-resolution microscopy^28^ methods.

In view of these findings, and taking into account that the cutoff distance we applied for the cluster analysis is comparable in size to the γH2AX objects segmented in the pseudo-wide-field images (Supplementary Fig. 3A), we conclude that γH2AX foci, as previously identified by conventional microscopy techniques, correspond to spatially organized clusters, composed of several distinct nano-foci of phosphorylated H2AX in close spatial proximity whose pattern in the nucleus depends on the progression of DDR. While clusters are chromatin higher-order organization units in the half-a-megabase-pair size range, nano-foci are lower-order chromatin organization units whose size spans 40–160 kb.

### γH2AX clusters contain single DNA DSBs

As previously reported, in higher eukaryotes^6^,^7^,^8^, γH2AX is proposed to spread up to Mbps from the lesion site in a non-homogenous non-symmetrical fashion^11^,^12^. This implies that γH2AX may also be found reasonably far from the actual DNA break. Indeed, on severe localized DNA damage (for example, caused by accelerated charged particles), pan-nuclear H2AX phosphorylation is promptly induced by ATM and DNA-PK^29^. It is then obvious that not all γH2AX-decorated chromatin contains a DNA DSB in the immediate vicinity.

Based on the linear increase of γH2AX nano-foci numbers, we observed up to 10 Gy (Supplementary Fig. 6G), and on the assumption that 1 Gy X-ray induce 30–55 DSBs per diploid human genome^30^,^31^,^32^,^33^, we estimated that 10 Gy X-ray would result in 470–860 DSBs in the ploidy-adjusted genome. Such numbers are conspicuously close to the number of γH2AX clusters we observed on IR (95% confidence interval of median cluster number at 0.5 h: 767–1,133; Fig. 6c and Supplementary Fig. 6H).

**Figure 7:**
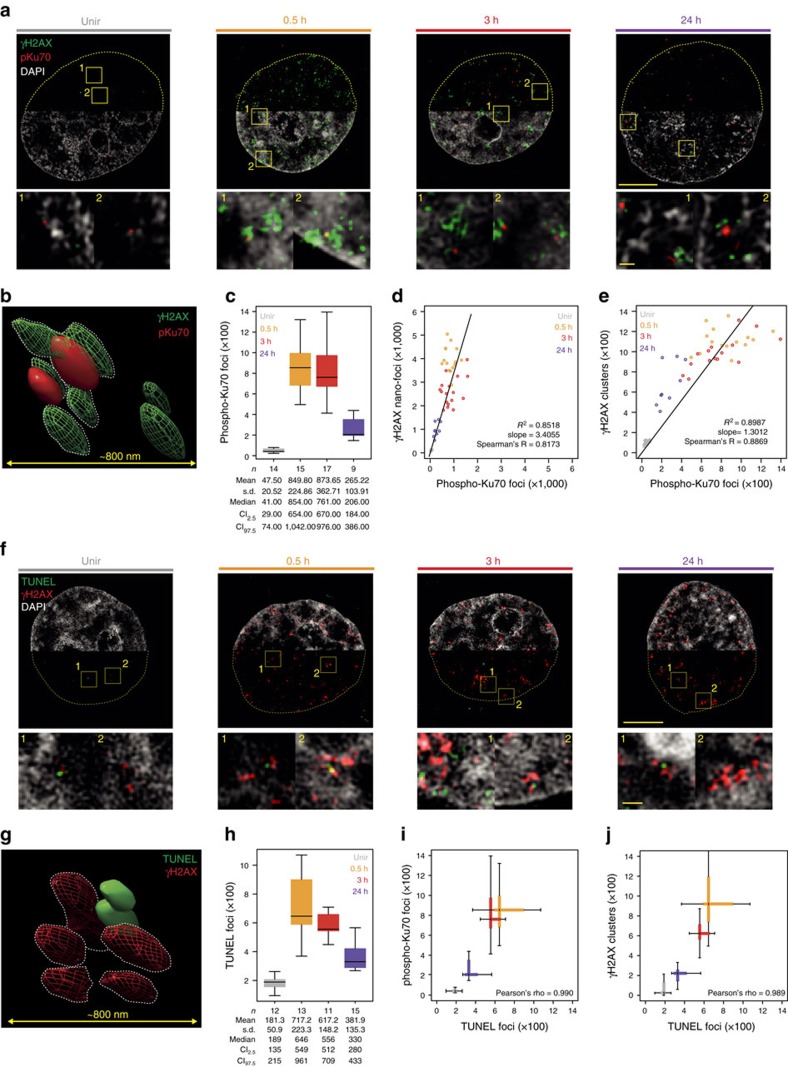
Single phospho-Ku70- or TUNEL-labelled DNA DSBs are embedded in γH2AX clusters. (**a**) Exemplary 3D-SIM images of γH2AX and phospho-ku70 immunofluorescence before and during DDR. Shown are the mid-nuclear section (top) and enlarged views from the yellow frames (bottom). (**b**) 3D rendering of γH2AX and phospho-Ku70 immunostaining, 24 h after IR. (**c**) Phospho-Ku70 foci number distributions before and during DDR, from 3D-SIM images (one-way ANOVA with Dunnett's correction: *P*<10^−3^). Scatter plots of phospho-Ku70 foci and γH2AX nano-foci (**d**) or γH2AX clusters (**e**). Each dot represents a single-cell nucleus. (**f**) Exemplary 3D-SIM images of γH2AX and TUNEL immunofluorescence before and during DDR. Shown are the mid-nuclear section (top) and enlarged views from the yellow frames (bottom). (**g**) 3D rendering of γH2AX and TUNEL immunostaining, 24 h after IR. (**h**) TUNEL foci number distributions before and during DDR, from 3D-SIM images (one-way ANOVA with Dunnett's correction: *P*<10^−3^). Comparison between TUNEL and phospho-Ku70 (**i**) or γH2AX clusters (**j**) distributions, before and during DDR (*P*<10^−3^). Scale bars, 5 μm and 500 nm for main micrographs and magnified regions, respectively. All boxes and whiskers are as in Fig. 1. *n*: number of analysed cells. Results are from two independent experiments.

To directly estimate the number of DNA DSBs before and during the DDR, we recorded 3D-SIM super-resolution images of immunofluorescently labelled phospho-Ku70 proteins, which are directly associated to the broken ends, together with γH2AX. As shown in Fig. 7a,b, most of the phospho-Ku70 signal was surrounded by several γH2AX nano-foci. Remarkably, the number of phospho-Ku70 focal structures matched with good agreement that of our previously measured clusters (Fig. 7c). Also, the slopes of the linear regression lines computed while fitting the number of phospho-Ku70 and γH2AX nano-foci or clusters indicate that we measured ∼3.4 γH2AX nano-foci per phospho-Ku70 focal structure, or in other words, that there are ∼1.3 phospho-Ku70 focal structures per γH2AX cluster (Fig. 7d,e). We observed similar results by assessing the number of DNA DSBs by terminal deoxynucleotidyl transferase dUTP nick end-labelling (TUNEL). TUNEL signal was often surrounded by several γH2AX nano-foci (Fig. 7f,g) and the number of TUNEL focal structures recapitulates the DDR (Fig. 7h). Finally, we observed a robust agreement between the numbers of TUNEL focal structures and phospho-Ku70 (Fig. 7i) or γH2AX clusters (Fig. 7j). Together, these data demonstrate that γH2AX clusters are γH2AX-decorated multi-unit chromatin structures containing a single DNA DSB.

### CTCF delimits phosphorylated H2AX chromatin domains

Altogether, the structural features we described about γH2AX clusters underpin the role of a structural organization factor in regulating their formation and kinetics. CTCF is involved in diverse cellular processes, including V(D)J recombination^34^, regulation of transcription^35^,^36^ and replication^17^. It mainly acts as a regulator of chromatin architecture^37^,^38^ by forming and keeping chromatin loops, and the presence of CTCF-binding motif close to the boundaries of large looping chromatin domains has been recently confirmed by *in situ* Hi-C^15^. In view of these observations, and based on CTCF insulating properties, we next investigated the relationship between CTCF and γH2AX levels during DDR.

**Figure 8:**
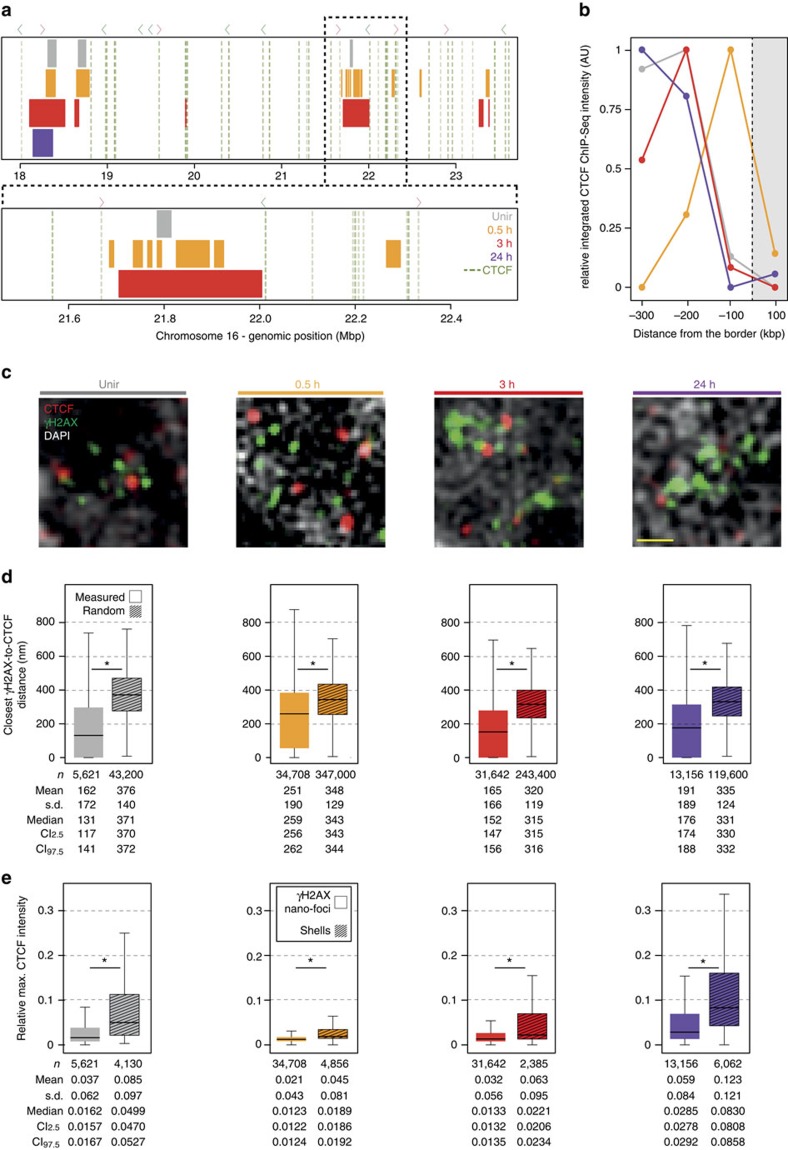
Genomic and microscopic analysis of CTCF spatial distribution in γH2AX-decorated chromatin. (**a**) Genomic localization of γH2AX ChIP-Seq domains (coloured bars) and CTCF genomic footprint (dashed green lines) in a representative region of chromosome 16. Dashed black line: magnification. Coloured arrowheads: orientation of CTCF-binding sites (red: forward; green: reverse). Details about γH2AX ChIP-Seq domains are in Supplementary Methods and Supplementary Fig. 4. ChIP-Seq CTCF profiles were retrieved from publicly available databases (UCSC Accession: Encode wgEH000080, wgEH000543, wgEH000401 and wgEH000470). (**b**) CTCF occupancy outside or inside γH2AX ChIP-Seq domains. The intensity of each CTCF peak in 100 kb bins upstream and downstream of the border of γH2AX ChIP-Seq domains (grey box) is summed and then presented as one-sided distribution. The bins range from ±300 to ±200, ±200 to ±100, ±100 to 0 and 0 to ±100 kb (inside the domain), with 0 being the border of each domain. AU: arbitrary unit. Genome-wide CTCF footprint localization relative to γH2AX ChIP-Seq domains' borders. For each domain, the distance in kb between its boundaries and the closest CTCF peak is measured and plotted as a bar (dashed lines). (**c**) Representative 3D-SIM images of immuno-stained γH2AX and CTCF before and during DDR. Scale bar, 500 nm. (**d**) Quantification of the closest centroid-to-centroid distance between CTCF and γH2AX nano-foci from 3D-SIM images. Measured (filled boxes) and simulated (patterned boxes) distances are shown. The latter were obtained from simulated random distributions of CTCF and γH2AX nano-foci (100 iterations). (**e**) Quantification of maximum CTCF intensity in γH2AX nano-foci and in surrounding shells. Maximum CTCF fluorescence in the segmented space normalized over the maximum CTCF fluorescence of the entire nucleus is plotted. All boxes and whiskers are as in Fig. 1. *n*: measured distances (**d**) or analysed shells (**e**) from two independent experiments. **d**,**e**: Mann–Whitney test: *P*<10^−3^.

We identified the genomic location of putative CTCF-binding sites, based on a consensus motif modified from previous studies^15^,^39^ (Supplementary Fig. 7A). The analysis resulted in 3,909 CTCF-binding sites, separated by a median intervening distance of ∼370 kb (IQD: 127–914 kb; Supplementary Fig. 7B). The orientation of CTCF motif had little to no impact on the measured distances (Supplementary Fig. 7B). This size range was comparable to that of γH2AX clusters rather than with that of single γH2AX nano-foci (Supplementary Fig. 7B), suggesting that individual clusters can be delimited by CTCF-binding sites. To validate such hypothesis at genomic level, we integrated our 3D-SIM-filtered γH2AX ChIP-Seq profiles (Supplementary Fig. 4) with publicly available HepG2 CTCF ChIP-Seq data. We identified ∼140,000 CTCF genomic footprints, including CTCF occupancy levels ranging from very low to very highy. Due to the inherent nature of this ChIP-Seq data, it is unlikely that all those CTCF peaks would actually be present at the same time in a given cell. Therefore, we focused our analysis only on those CTCF genomic footprints whose occupancy score was maximum, assuming these sites would be conserved among different cell types. This reduced the number of CTCF footprints to 5,322. Remarkably, these sites were flanking most of the genomic γH2AX domains, before and during the DDR (Fig. 8a), yet the two signals seldom overlapped. In addition, CTCF ChIP-Seq signal intensity (that is, CTCF abundance) was higher upstream or downstream of the borders of each γH2AX genomic domain than that computed inside the domain (Fig. 8b), indicating that high-occupancy CTCF sites function as barriers for γH2AX spreading.

Next, we investigated the 3D-distribution of γH2AX and CTCF before and during DDR at single-cell level by 3D-SIM. On IR, CTCF foci were often in the immediate proximity of γH2AX nano-foci (Fig. 8c and Supplementary Fig. 7C,D). The majority (∼75%) of the centroid-to-centroid distances between each γH2AX nano-focus and the closest CTCF focal structure were within 400 nm, and starting from 3 h post infrared, they all were below 200 nm (Fig. 8d). In all cases, the measured distances were smaller than distances between simulated random objects whose populations were comparable in numbers to those of CTCF and γH2AX nano-foci at each stage of DDR (Fig. 8d and Supplementary Fig. 7E). Because γH2AX nano-foci in our 3D-SIM images have a radius of ∼100 nm, and CTCF focal structures showed comparable size, our results imply that the two objects would thus be in tight contact, with CTCF focal structures flanking γH2AX nano-foci. On exposure to IR, and based on the higher CTCF density in GC-rich regions, the expected γH2AX-to-CTCF distance should be equal to, if not shorter than, that we observed in the control sample (Fig. 8d, Unir, median: 131 nm). However, 0.5 h post IR, the median γH2AX-to-CTCF distance was two times longer (259 nm). Moreover, during the late stage of the DDR, the majority of DSBs were associated to heterochromatic regions (with lower GC content). In these regions, CTCF density is lower (compared with euchromatin) and the expected γH2AX-to-CTCF distance should be equal to, if not longer than, that we measured in a random distribution. Yet, the observed median γH2AX-to-CTCF distance was only half of that we obtained from a random distribution (Fig. 8d, 24 h measured: 176 nm; 24 h random: 331 nm). Such close spatial proximity was confirmed by the observation that CTCF signal was more abundant in the surroundings of γH2AX nano-foci (as measured in the previously described shells) rather than overlapping with them (Fig. 8e and Supplementary Fig. 7F).

Taken together, our genomic and microscopy data strongly support that CTCF delimits γH2AX chromatin, and the two are in close spatial proximity.

### CTCF is critical for spatial regulation of γH2AX chromatin

**Figure 9:**
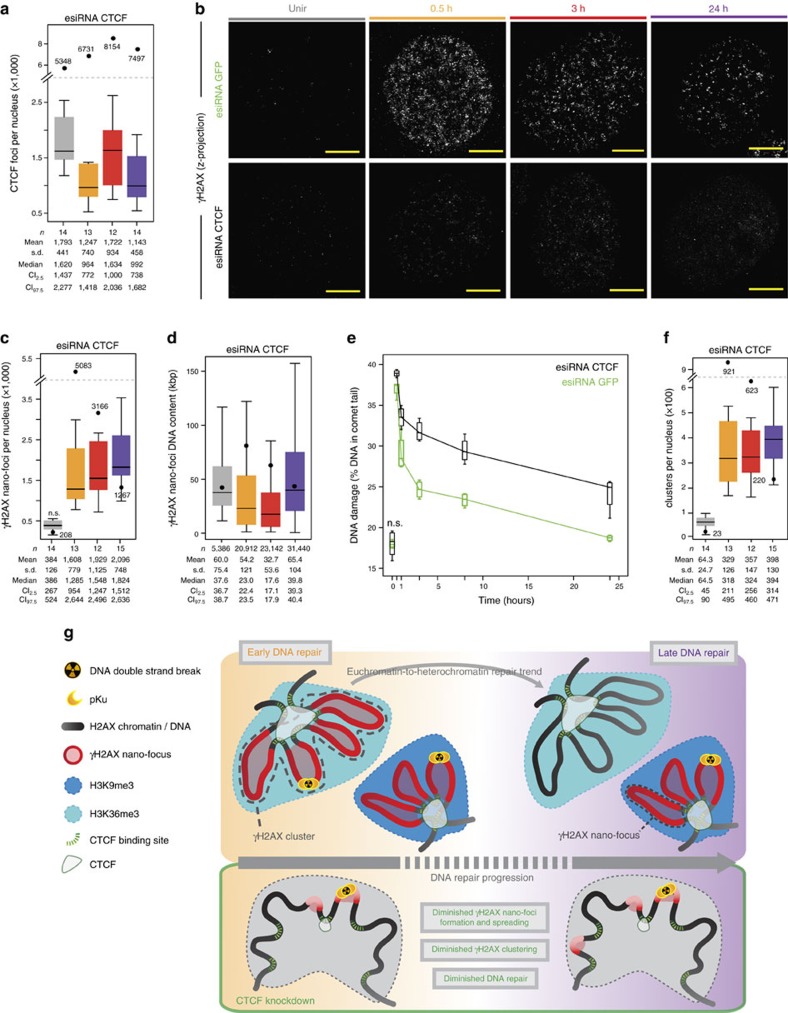
CTCF depletion inhibits γH2AX nano-foci and cluster formation and diminishes the DNA repair capability. (**a**) Number of CTCF foci in esiRNA-depleted cells before and during DDR. Black dots: median number of CTCF foci in wild-type cells. (**b**) Impairment of γH2AX nano-foci and 3D-clusters formation during DDR as assessed by immunofluorescence of 3D-SIM images in CTCF-depleted cells. Scale bar, 5 μm. (**c**) γH2AX nano-foci number distributions before and after IR, in CTCF siRNA-treated cells. Black dots: median number of γH2AX nano-foci of untreated cells (from Fig. 1).NS: two-tailed *t*-test, *P*>0.05. (**d**) γH2AX nano-foci DNA content distributions before and after IR, in CTCF siRNA-treated cells. Black dots: median DNA content of γH2AX nano-foci of untreated cells (from Fig. 2). (**e**) DNA fragmentation measured by the neutral comet assay. Boxes represent the mean of medians from four replicates (two biological replicates in duplicate), each consisting of 60 comet measurements. NS: not significant (*t*-test, *P*>0.05). (**f**) γH2AX cluster distributions before and after IR, in CTCF siRNA-treated cells. Black dots: median number of γH2AX clusters in untreated cells (from Fig. 6). All boxes and whiskers are as in Fig. 1. Comparisons between time points (one-way ANOVA with Dunnett's correction) or between esiRNA-treated and wild-type cells (Wilcoxon/Mann–Whitney rank sum) are all statistically significant unless otherwise specified. (**g**) Model for cluster special arrangement during DDR, showing the time-dependent euchromatin-to-heterochromatin repair trend (top) and how γH2AX spreading is hampered by CTCF depletion with the concomitant loss of 3D-arrangement of chromatin loops (bottom).

Finally, we investigated whether the perturbation of CTCF levels would affect the spatial distribution of γH2AX-decorated chromatin. While CTCF knockout is lethal, a number of studies have shown neither effects on the cellular and nuclear morphology, nor in the cell cycle progression up to 72 h post CTCF knockdown^40^,^41^. In our experimental system, esiRNA-mediated CTCF depletion to ∼40% of the control protein levels (Supplementary Fig. 8A,B), resulted in a mild radiosensitization (∼20%; Supplementary Fig. 8C) and a coherent decrease (70–85%) of CTCF foci in 3D-SIM micrographs, before and during DDR (Fig. 9a). Notably, CTCF depletion strongly impaired the formation of γH2AX nano-foci (Fig. 9b), which were smaller, diminished in numbers, and presented decreased volume and DNA content (Fig. 9c,d and Supplementary Fig. 8D,E). Only at 24 h post IR, the number of γH2AX nano-foci was comparable to that of the mock-knockdown samples, although with decreased fluorescence intensity, indicating a defect in the activation of the DDR. Indeed, CTCF-depleted cells showed a diminished DNA repair capability as assayed by comet single-cell analysis (Fig. 9e). Such defect was more prominent at the mid and late stages of DDR, suggesting that optimal CTCF levels are required to mount an efficient DDR. In this context, CTCF role in chromatin structural regulation may be crucial. Overall, the diminished γH2AX response resulted in a ∼2.9-fold decrease in cluster formation (Fig. 9f). Remarkably, ATM and DNA-PKcs, the main signalling effectors involved in H2AX phosphorylation, were promptly activated on IR in both mock- and CTCF-depleted cells (Supplementary Fig. 9A,B), indicating that the presence of functional key factors of the DDR is necessary but not sufficient to trigger a proper response to DNA damage. In conclusion, we propose that CTCF, by preserving the 3D organization of the chromatin, is critical for the activation of an efficient DDR and, in such context, it functions as a regulator of the structural component of DDR.

## Discussion

In this study, the use of high prospecting super-resolution light microscopy technologies enabled us to identify the elementary structural units read by the DNA repair machinery, analysed as γH2AX focal structures following the exposure to IR. The γH2AX nano-foci we identified are two- to threefold smaller—with lateral diameters of ∼200 nm—and contain ∼10% of the conventionally estimated Mbp DNA content^42^. Similar γH2AX substructures sizes were recently measured after heavy ion irradiation^43^, despite the highly ionizing power charged particles possess, thus further supporting our findings.

Importantly, γH2AX nano-foci form clusters of approximately four chromatin units, and each cluster, rather than each of its structural components, contains one DSB, assessed by direct DNA end-labelling or by the presence of phospho-Ku70. This is supported by the good agreement between the predicted number of DSBs induced by the dose of IR employed in this work and the numbers of γH2AX clusters in control cells. γH2AX clusters are spatially distributed in the nuclear space according to a pattern that is dependent on the progression of DDR. Such pattern recapitulates the previously described repair kinetics, underlining an euchromatin-to-heterochromatin repair trend, which is likely dictated by the chromatin compaction state: chromatin regions that were already in an open state (for example, marked by H3K36me3) would be repaired earlier, while compact chromatin requires further structural remodelling before the DNA repair machinery could eventually exert its activity (Fig. 9g), For the latter, actual DNA decondensation, assessed as decrease of DAPI intensity, occurred while maintaining the main local histone modification (for example, H3K9me3), thus uncoupling DNA compaction from histone modifications. While chromatin relaxation seems to be dispensable for the DNA repair to occur at pericentromeric heterochromatin^44^, we propose that the uncoupling of chromatin modifications and the actual chromatin decondensation is crucial to reestablish the original chromatin structure once DNA repair is accomplished.

In our 3D-SIM images, γH2AX clusters presented a discontinuous phosphorylation pattern, with γH2AX and CTCF showing mutually exclusive signals, although the two were in close spatial proximity. However, not all γH2AX nano-foci presented proximal CTCF foci. The latter likely consist of more than one CTCF molecule, and their detection may be influenced by a variety of factors, such as the CTCF-binding site density, differences in the binding affinity^45^ of such sites and CTCF protein levels. It is tempting to speculate that the discontinuously phosphorylated pattern we observed is due to the presence of multiple CTCF molecules bound to their cognate consensus sequences but not resolvable by our imaging techniques. To discriminate between each individual chromatin loop bound by a pair of CTCF molecules, would demand single molecule sensitivity *in situ* 3D methods. Nonetheless, it is equally possible that other chromatin structure regulators (for example, cohesion complex^12^), histone turnover (for example, during DNA repair^46^) as well as biological processes such as transcription^11^ antagonizing γH2AX formation and/or spreading along the chromosome contribute to the discontinuously phosphorylated pattern.

Finally, we show that CTCF has a critical role in the formation and spatial clustering of γH2AX nano-foci. CTCF-depleted cells present less γH2AX nano-foci, which are smaller and contain less DNA than those we observed in mock-treated cells. As a consequence, the DDR is delayed and the repair capability is diminished, despite the efficient activation of the main signalling effectors involved in H2AX phosphorylation (for example, DNA-PKcs or ATM). This indicates that a structural organization impairment—caused by CTCF depletion—results in a poor DDR. On CTCF depletion, the frequency of interactions of CTCF molecules with one another is decreased, leading to a diminished loop formation and a more sparse (that is, non-clustered) distribution of γH2AX nano-foci (Fig. 9b–g). Overall, this scenario emphasizes the need for a (dynamically) regulated 3D organization of the chromatin, whereby the 3D spatial proximity of chromatin loops could boost the local processivity of the committed kinases and assure an efficient DDR. In such context, because the CTCF-knocked-down cells display similar numbers of γH2AX nano-foci to the number of nano-foci cluster in control cells, we propose that in the absence of CTCF, spreading of γH2AX is impaired and, thus, this mark is restricted to the vicinity of the DSBs, that is, within one nano-focus (Fig. 9g).

In conclusion, our study demonstrates that the decreased levels of a single structural factor (CTCF), accounting for the (dynamic) stability of chromatin, *per se* dramatically hinder γH2AX spreading. While it is likely that additional factors (for example, DNA and histone methylation readers) contribute to this process, namely at heterochromatic regions, we propose that CTCF functions as a regulator of the structural component of DDR, preserving a crucial (dynamic) 3D organization of the chromatin and, thus, enabling an efficient DDR.

## Methods

### Cell culture and irradiation

Cervical carcinoma HeLa cells (ATCC No. CCL-2) cells were used throughout the study. A single exposure to 10 Gy X-ray was applied (250 kV, 16 mA, 2.5 Gy min^−1^ – GE Isovolt Titan) to induce DNA damage and trigger DDR. On exposure to IR, cells were incubated in a humidified environment, with 5% CO_2_ at 37 °C as indicated. Sham-irradiated control cells were included. C2C12 (ATCC No CRL-1772) cells were used for CRISPR-Cas9 experiments. HeLa and C2C12 cells were cultured in DMEM (4.5 g l^−1^ glucose, Biochrom AG) supplemented with 10% and 20% fetal calf serum (Biochrom AG), respectively. All media were supplemented with 2 mM L-glutamine (Sigma), 100 U per ml penicillin and 100 μg ml^−1^ streptomycin (Sigma). All cell lines were tested for mycoplasma and found free of contamination (MycoAlert, Lonza).

### Growth curve and cell cycle distribution

Cells were seeded 24 h before exposure to IR. After IR, cells were incubated for indicated times, before trypsinization and count with a coulter counter, in triplicates. The remaining cells were then fixed in 2% formaldehyde, permeabilized for 8 min with 0.5% Triton X-100 in PBS, stained with DAPI (1 μg ml^−1^) and analysed at the flow cytometer Partec PAS III system (Partec) for cell cycle distribution. Data were analysed with FlowJo software (Tree Star, Inc.).

### Apoptosis assay

To detect apoptosis, TUNEL assay was performed according to the manufacturer's instructions (Roche, #11684795910) and a minimum of 1,000 cells was scored by microscopy in two independent experiments.

### Spectral karyotyping

Cells were treated with colcemid (0.1 μg ml^−1^; Invitrogen, Darmstadt, Germany) 2 h before collecting to accumulate metaphase cells. Chromosome preparations were made according to standard procedures and hybridized with the 24XCyte Multicolor FISH Probe Kit (MetaSystems, Altlussheim, Germany). Metaphase spreads were examined with an Axio Imager Z1 microscope (Zeiss, Oberkochen, Germany) equipped with appropriate filter sets. At least 100 images of metaphases were taken, further processed using ISIS software (MetaSystems) and analysed to produce the karyotype.

### CTCF knockdown

A number of 10^5^ cells were transfected with 15 nM of a esiRNA pool (Sigma-Aldrich) using HiPerfect (Qiagen). The CTCF esiRNA is corresponding to the region 692–1195 of the human CTCF transcript (NM_006565.3). For mock treatments, cells were transfected using an esiRNA pool (Sigma-Aldrich) targeting the *GFP* gene. Cells were incubated 24–96 h post transfection and knockdown efficiency was monitored every 24 h.

### Immunoblotting

Whole-cell extracts were prepared by freeze and thaw lysis (three cycles) in 600 mM NaCl, 20 mM Tris-HCl pH 7.8, 20% glycerol. After SDS–PAGE, proteins were transferred onto PVDF membrane in semi-dry conditions. The membrane was then blocked in 5% non-fat dry milk buffer and incubated with mouse anti-γH2AX (Clone JBW301, Upstate, 1:5,000). Immunoblots were stained with corresponding HRP-conjugated secondary antibodies (GE Healthcare, 1:20,000) and detected with the enhanced chemiluminescence detection system (Amersham Biosciences). Quantification was performed using ImageJ.

For the validation of antibody specificity and cross-reactivity, a dilution series of synthetic peptides (CKATQASQEY; Peptide Specialty Laboratories GmbH), with the underlined serine in either phosphorylated or non-phosphorylated form, was immobilized on a nitrocellulose membrane at the indicated concentrations and probed with anti-γH2AX and anti-H2AX as described above.

CTCF knockdown western blots were developed using a rabbit anti-CTCF (#D31H2, Cell Signaling, 1:700) and a mouse anti-actin (AC-40, Sigma-Aldrich, 1:1,000) and overnight incubation at 4 °C, followed by a direct immunofluorescence detection using anti-rabbit-IgG-Cy5 (#711-175-152, Jackson, 1:1,000) and an anti-mouse-IgG-Alexa488 (A11029, Invitrogen, 1:1,000). Images were recorded using a AI600 Imager (Amersham) and quantified using ImageJ.

### Immunofluorescence

Cells were fixed in 3.7% formaldehyde and permeabilized in 0.5% Triton X-100 in PBS at room temperature (RT). The following primary antibodies were used: mouse anti-γH2AX (Clone JBW301, 1:500, Upstate), rabbit anti-H3K9me3 (#07–422, Upstate, 1:500), rabbit anti-H3K9me3 (#39161, Active Motif, 1:500), rabbit anti-H3K36me3 (ab9050, Abcam, 1:2,000); rabbit anti-phospho-Ku70 (pS5) (#ab61783, Abcam, 1:400); mouse anti-phospho-ATM (pS1981) (#MAB3806, Millipore, 1:100); rabbit anti-phospho-DNA-PKcs (pS2056) (#ab18192, Abcam, 1:100) and rabbit anti-CTCF (#2899, Cell Signaling, 1:900). For phospho-Ku70 detection cells were prefixed in 1% formaldehyde and then extracted with 0.7% Triton X-100 two times by 5 min^47^ and subsequently fixed in 3.7% formaldehyde. Antibody incubation was performed at 4 °C over night in 1% BSA in PBS. For CLSM and 3D-SIM, signals were detected with goat anti-mouse-IgG-AlexaFluor 488, goat anti-rabbit-IgG-AlexaFluor 594 (1:800, Invitrogen), donkey anti-mouse-IgG-AlexaFluor 488 (A-21202, Thermo Fisher Scientific, 1:400), donkey anti-rabbit-IgG-AlexaFluor 594 (A-21207, Thermo Fisher Scientific, 1:400). For STED, γH2AX was detected with goat anti-mouse-IgG STAR 635P (#2-0002-007-5, Abberior, 1:100) or goat anti-mouse-IgG STAR 580 (#2-0002-005-1, Abberior, 1:100). DNA was counterstained with 36 nM DAPI (for 3D-SIM), 1 μM propidium iodide (confocal microscopy) or 2.5 μM SiR-DNA (Spirochrome), before cells were mounted with Vectashield antifade medium (Vectorlabs).

### CRISPR-Cas9 targeting to heterochromatic major satellite DNA

Subconfluent C2C12 cells were transfected with Cas9 (pCMV-hCas9, Addgene ID: 41815) and major satellite gRNAs (U6-MaSgRNA) by means of Lipofectamine 3000 (Thermo Fisher Scientific) according to the manufacturer's instructions. Cells were then fixed in 3.7% formaldehyde for 10 min and immunofluorescence followed (as described above).

### DNA DSB detection by TUNEL assay

Cells were grown and irradiated as described above. At the indicated time points, cells were fixed in 3.7% paraformaldehyde for 10 min. The fixation was quenched with 125 mM glycine in PBS for 10 min. Fixed cells where permeabilized in 0.5% Triton X-100 for 20 min, and equilibrated for 10 min in blunting buffer (100 mM Tris-HCl, 50 mM NaCl, 10 mM MgCl_2_, 0.025% Triton X-100 and 5 mM DTT, pH 7.5). End repair was performed using 4 μl T4 polymerase (NEB: M0203S 3,000 units ml^−1^) and 4 μl T4 polynucleotide kinase (NEB: M0201S 10,000 units ml^−1^) in 82 μl blunting buffer, supplemented with 10 μl 1 mM dNTPs for 45 min. Slides were then equilibrated in TdT buffer for 10 min and the TUNEL reaction was performed according to the ‘In Situ Cell Death Detection Kit' (Roche) with Fluorescein modified dUTPs, for 4 h at 37 °C according to the manufacturer's instructions. Following the TUNEL reaction, cells were blocked in 1% BSA in PBS for 20 min. γH2AX staining was performed as described above. Incorporated fluorescein-dUTPs were detected by a rabbit anti-FITC (CUSABIO, 1:500) and a anti-rabbit-IgG Alexa488 secondary antibody (Jackson ImmunoResearch, 1:800). All steps were conducted at RT, unless otherwise specified.

### Comet assay

DNA repair kinetics in CTCF knockdown cells were measured using the neutral comet assay. In brief, CTCF was depleted as described above and 72 h post esiRNA transfection, the cells were exposed to 10 Gy X-ray. At the indicated time points, cells were trypsinized and 2 × 10^5^ cells ml^−1^ were embedded in 0.8% low-melting point agarose (Sigma type VII). Lysis was performed for 4 h at 4 °C in lysis buffer (10 mM Tris, 150 mM NaCl, 1% N-lauryl-sarcosinate, 1% Triton X-100, 0.5% DMSO, pH 8.0) and electrophoresis was done in 1 × TBE at 4 °C (1 V cm^−1^) for 25 min. Slides were then dehydrated in 70% ethanol and rehydrated in staining buffer (TBE supplemented with SybrGreen, 1:10,000) to stain the DNA^48^. Two biological replicates (in duplicates) were performed and 60 comets per slide were scored using Komet 4 (Kinetic Imaging Ltd.).

### Microscopy

Confocal microscopy images were acquired using a Spinning Disk microscope (Perkin Elmer Vox1000) equipped with a × 60 NA 1.4 oil immersion lens (CFI Apochromat TIRF), with a pixel size of 120 nm or with a Leica TCS SP5 confocal microscope using a Plan Apo × 63 NA 1.4 oil immersion objective. Cells were recorded as z-stacks with a z-spacing of 0.2 μm.

Super-resolution microscopy images were acquired using a 3D structured illumination microscope (DeltaVision OMX V3, GE Healthcare) and a 2C STED 775 QUAD Scan microscope (Abberior Instruments). 3D-SIM was performed with a × 100 NA 1.4 objective lens with a pixel size of 39 nm and a z-spacing of 125 nm (ref. 18). STED was performed with a × 100 NA 1.4 Olympus UPlanSApo objective lens with a pixel size of 20 nm and excitation lasers of 488, 594 or 640 nm, and a 775 nm depletion laser.

High-content imaging was performed using the Operetta system (Perkin Elmer). Samples were imaged using a × 20 NA 0.45 air objective with three planes of 1 μm spacing, using the following filters: DAPI: excitation wavelength (ex): 360–400 nm, emission wavelength (em): 420–480 nm; Alexa488: ex: 460–490 nm, em: 500–550 nm; Alexa594: ex: 560–580 nm, em: 590–640 nm.

### Image analysis

For confocal microscopy, the images were analysed in ImageJ using the nuclear staining as a mask to measure the total intensity of the γH2AX signal per nucleus. Foci were scored in 3D using Volocity (Perkin Elmer) by the following workflow: find objects (nucleus), threshold automatic, size minimum 500 μm^3^; find foci: threshold 4,000 constant for pseudo-wide-field and 5,000 for deconvolved images, respectively. Minimum size: 0.05 μm^3^, followed by ‘separate touching objects' with a guide size of 0.5 μm^3^. Different thresholds were applied, because pseudo-wide-field and deconvolved images are in different bit depth. All counts were double-checked by manual counting of randomly chosen samples by at least three experimenters.

For CRISPR-Cas9 experiments, confocal images of C2C12 cells were segmented into background, nuclei and chromocentres by pixel-wise classification via supervised machine learning (default Random Forest classifier and pixel features from the Trainable Weka Segmentation plugin in Fiji). The classifier was trained on manually labelled pixels of the DAPI channel in one image and then applied to all images. For each image, mean intensities in the H3K9me3 and γH2AX channels were determined for each chromocentre object (>100 px^2^) within the largest object in the nuclear mask. To analyse DNA decondensation at repair sites in CRISPR-Cas9 experiments STED images of C2C12 cells were segmented into background, nuclei and chromocentres by pixel-wise classification as described above for confocal images. The classifier was trained on manually labelled pixels of the SiR-DNA channel in one image and then applied to all images (each image's pixel intensity range was mapped to the 8-bit range to account for differences in staining intensities). For each image, the circularity of chromocentre objects (>100 px^2^) within the nucleus was determined. Three rounds of binary erosion with a 3 × 3 px-box followed by three rounds of binary dilation were applied to the segmentation results to smooth the borders of segmented objects.

3D-SIM images were exported from the DeltaVision software (softWoRx 6.0 Beta 19, Applied Precision) and converted to 16-bit images per channel. Foci counting was done using Volocity 6.3 (Perkin Elmer) or with the 3D foci picker plugin in ImageJ (imagej.nih.gov/ij/). Nearly identical results were obtained and the numbers from Volocity were used. In detail, the individual z-sections were imported and merged to a volume with the above-mentioned pixel sizes and z-spacing. First, the nucleus was identified by setting a manual threshold and a lower volume limit of 200 μm^3^ followed by a ‘Fill in Holes' step and two iterations of ‘Dilate' and ‘Close' to fill in all the DAPI weak volumes. The intensities and voxel coordinates of the whole nucleus were registered. Next, the γH2AX and H3K36me3 or H3K9me3 foci were identified with a lower threshold of 1,000 and a minimum object size of 0.001 μm^3^. To separate close spaced objects, a final ‘Separate Touching Objects' step with a nominal volume of 0.05 μm^3^ was used. The foci identified were restricted to the previously defined nuclear volume to remove possible unspecific signals from outside of the nucleus.

3D-SIM pseudo-wide-field imaging: after sample acquisition, the pseudo-wide-field images were calculated using softWoRx 6.0 Beta 19 according to the following workflow: the raw data from each 3D-SIM image z-stack was subdivided to isolate the first angle of acquisition. To this purpose, the maximum number of z-sections in each individual stack is divided by three. Then the projected five grid shifted section is averaged per z-position and colour channel. After that, the voxel dimensions are adjusted from 0.625 to 0.125 μm in the z-dimension by adjusting the file headers. The alignment of the new stack was done with the parameters used for 3D-SIM reconstruction. The following parameters of the softWoRx software were used: normalize intensity, use photosensor, correct bleaching, replace z-lines and smooth z-lines. To reverse the optical distortion in the images, the aligned 3D stack was deconvolved with the instrument-specific optical transfer function (OTF) with the following settings: ‘enhanced ratio (aggressive)' and ‘noise filtering medium'.

For CTCF distance analysis, the previously described protocol was extended as follows: CTCF domains detection was restricted to the nuclear volume, with an automated threshold and a minimum size of 0.001 μm^3^. Then, the segmented γH2AX nano-foci were extended in all dimensions by three voxels (117 × 117 × 375 nm) and the resulting γH2AX nano-foci volume was subtracted to obtain the γH2AX foci shells. Finally, the Euclidian distances between each γH2AX nano-focus and the closest CTCF domain were measured. All identified foci with the corresponding 3D coordinates and intensities for all recorded channels were exported and post-processed in R^49^. ImageJ and UCSF chimera^50^ were used for image visualization and 3D rendering, respectively. Simulations of CTCF and γH2AX distributions were run under R, using rgl and sphereplot packages. Hundred simulations of a sphere matching the average nuclear size of cells were run per time point. Every simulation contained objects whose numbers matched CTCF and γH2AX foci we recorded in 3D-SIM images.

For STED images, object dimensions (for example, diameters) were measured by manual object segmentation of randomly selected foci in ImageJ, using the analyse particle tool. For high-content images, analysis was performed using Harmony software (Perkin Elmer) with the following workflow: maximum projection of the planes, flatfield correction, find nuclei in DAPI channel, method M, splitting coefficient 0.1, general threshold 0.4 and guide size of 15 μm in diameter. Calculate intensity and morphology parameters for the nuclei. Discard nuclei touching the border, smaller than 100 μm^2^ and larger than 350 μm^2^. Filter nuclei for roundness >0.83 and with a 4 px Haralick contrast >0.8 and a DAPI signal CV of less than 30%. Measure the mean and integrated intensity for DAPI, γH2AX and CTCF in the selected nuclei areas.

### ChIP

Cells were fixed with 1% formaldehyde for 10 min at RT and cross-link was quenched with 125 mM glycine (5 min at RT). Nuclei were isolated after mild lysis in hypotonic buffer (10 mM HEPES pH 8, 1.5 mM MgCl_2_, 60 mM KCl) and 20 strokes in a tight dounce homogenizer. Chromatin was sheared in sonication buffer (0.5% SDS, 10 mM EDTA, 50 mM Tris-HCl pH 8.1). Fragmentation of chromatin was carried out by ultrasound treatment (Bioruptor UCD200) so that fragments of 200–300 bp length were obtained. Chromatin from 1 × 10^6^–2 × 10^6^ cells was immunoprecipitated with anti-γH2AX (Clone JBW301, Upstate, 3 μg) antibody. Chromatin was then incubated ON at 4 °C with protein G-coated magnetic beads (ChIP-IT Express, Active Motif). The collected chromatin (ChIP sample) was then reverse-crosslinked in the presence of 200 mM NaCl at 65 °C for at least 5 h, followed by RNase A (50 μg ml^−1^) treatment for 30 min at 37 °C and proteinase K (100 μg ml^−1^) treatment for 3 h at 50 °C. DNA elution was carried out in 1% SDS, 100 mM NaHCO_3_, in a rotary shaker at RT for 15 min. Pure DNA was isolated using the Qiagen PCR purification kit and 15–30 ng of size selected DNA fragments (Qubit fluorometric quantification) were used to produce ChIP-seq libraries (Illumina ChIP-Seq DNA sample Prep Kit). Input sample was essentially prepared following the same protocol, but the immunoprecipitation step was skipped.

### Next-generation sequencing and data analyses

ChIP-Seq libraries were processed through a high-throughput sequencing pipeline (Illumina Genome Analyzer II). Reads were mapped to the human genome (University of California, Santa Cruz (UCSC) hg19 assembly, based on the National Center for Biotechnology Information (NCBI) build 37.1) by means of SOAP2 software^51^, allowing up to two mismatches for each 36 bp read. All data sets were deposited in the Gene Expression Omnibus database (accession number: GSE60526). All γH2AX ChIP-Seq tracks were smoothed with a moving average of five intervals before further analysis. Genomic features and correlation analysis: all genomic features data were retrieved from publicly available databases (UCSC) (Supplementary Table 3). Most of the data were generated in HepG2 cells, but not all. Data that were originally generated in the hg18 assembly were transposed to hg19 using LiftOver (http://genome.ucsc.edu/cgi-bin/hgLiftOver). Reads per kilobase per million reads (RPKM)^52^ were calculated for non-overlapping 10 kb genomic intervals for all sequence tracks. The features were further normalized to the corresponding genome-wide average and correlation with γH2AX tracks was performed (Spearman's *ρ* correlation coefficient with *P*<2.2 × 10^−16^ in all cases).

### Statistical analysis

Overall, sample size was chosen so that groups (for example, time points) had comparable numbers (for example, number of imaged cells), whenever possible. High-content microscopy and next-generation sequencing provided large data sets ensuring statistical significance. All statistical analysis has been performed using R or GraphPad Prism. Briefly, in case data were normally distributed (Shapiro–Wilk test), ANOVA or Student's *t*-test were performed for groups or pairs, respectively. Else, Kruskal–Wallis or Wilcoxon/Mann–Whitney rank sum tests were used for groups or pairs, respectively.

### Integration of 3D-SIM and ChIP-Seq data

To integrate the ChIP-Seq data with 3D-SIM information, we first generated 25 independent profiles by applying a smoothing factor to each γH2AX ChIP-Seq data set (Supplementary Fig. 4A). Such smoothing factor is a moving average ranging from 1 (no smoothing) to 25 genomic intervals (indicated as ‘1D', in Supplementary Fig. 4A). In parallel, we measured the volume fraction occupied by γH2AX nano-foci as well as their corresponding DNA content, before and during the DDR (Supplementary Fig. 4B). In response to ionizing radiation, we observed an increase of the mean γH2AX-occupied nuclear volume (from 0.21±0.21% to 7.81±3.19%), which recapitulated the DDR (the volume was reduced to 3.70±1.39% and 0.66±0.43%, at 3 h or 24 h post-ionizing radiation, respectively). Next, we applied the mean volume fractions (0.21%, 7.81%, 3.70% and 0.66% for unirradiated, 0.5 h, 3 h and 24 h, respectively) to filter the previously smoothed genomic γH2AX ChIP-Seq data so that only the 10 kb genomic intervals from the top percentiles of the read density distributions were retrieved (Supplementary Fig. 4C). For example, as for the unirradiated cells, we sampled the 99.79th percentile (top 100–0.21%) of the intervals, while for the 0.5 h time point, we sampled the 92.19th percentile (top 100–7.81%) of the total RPKM γH2AX ChIP-Seq distribution. A representative image of filtered ChIP-Seq profiles is shown in Supplementary Fig. 4D. By applying these imaging-based thresholds, we obtained a linear coverage of 4.7 Mbp, 159.0 Mbp, 92.3 Mbp and 21.8 Mbp, at unirradiated, 0.5 h, 3 h and 24 h time points, respectively (Supplementary Fig. 4E). Finally, we employed the numbers of 3D γH2AX nano-foci to match the numbers of 1D nano-domains as follows: first, the number of 3D γH2AX nano-foci before and after the DDR was scaled down to the haploid genome size to match the genomic data (ploidy correction factor: HeLa_genome size_/haploid reference_genome size_=3.12); next, we chose the smoothing factor at which the number of γH2AX nano-foci and the number of retrieved genomic intervals matched best, at any given time point (Supplementary Fig. 4A, over-imposed crosses). All ChIP-Seq domains identified via such approach are referred to as ‘1D domains' and an estimate of the 1D domain size distribution is presented in Supplementary Fig. 4F.

### Data availability

Next-generation sequencing results are available at GEO (https://www.ncbi.nlm.nih.gov/geo/) under the accession number GSE60526. Other data that support the findings of this study are available from the corresponding author on reasonable request.

## Additional information

**How to cite this article:** Natale, F. *et al*. Identification of the elementary structural units of the DNA damage response. *Nat. Commun.* **8,** 15760 doi: 10.1038/ncomms15760 (2017).

**Publisher's note:** Springer Nature remains neutral with regard to jurisdictional claims in published maps and institutional affiliations.

## Supplementary Material

Supplementary Information Supplementary Figures, Supplementary Tables, Supplementary Methods, and Supplementary References
