# Supplementary material for: Identification of the elementary structural units of the DNA damage response
Source: Nat Commun. 2017 Jun 12;8:15760. doi: 10.1038/ncomms15760 (PMC5472794; doi:10.1038/ncomms15760)
Supplement: Supplementary Information — Supplementary Figures, Supplementary Tables, Supplementary Methods, and Supplementary References [file ncomms15760-s1.pdf]

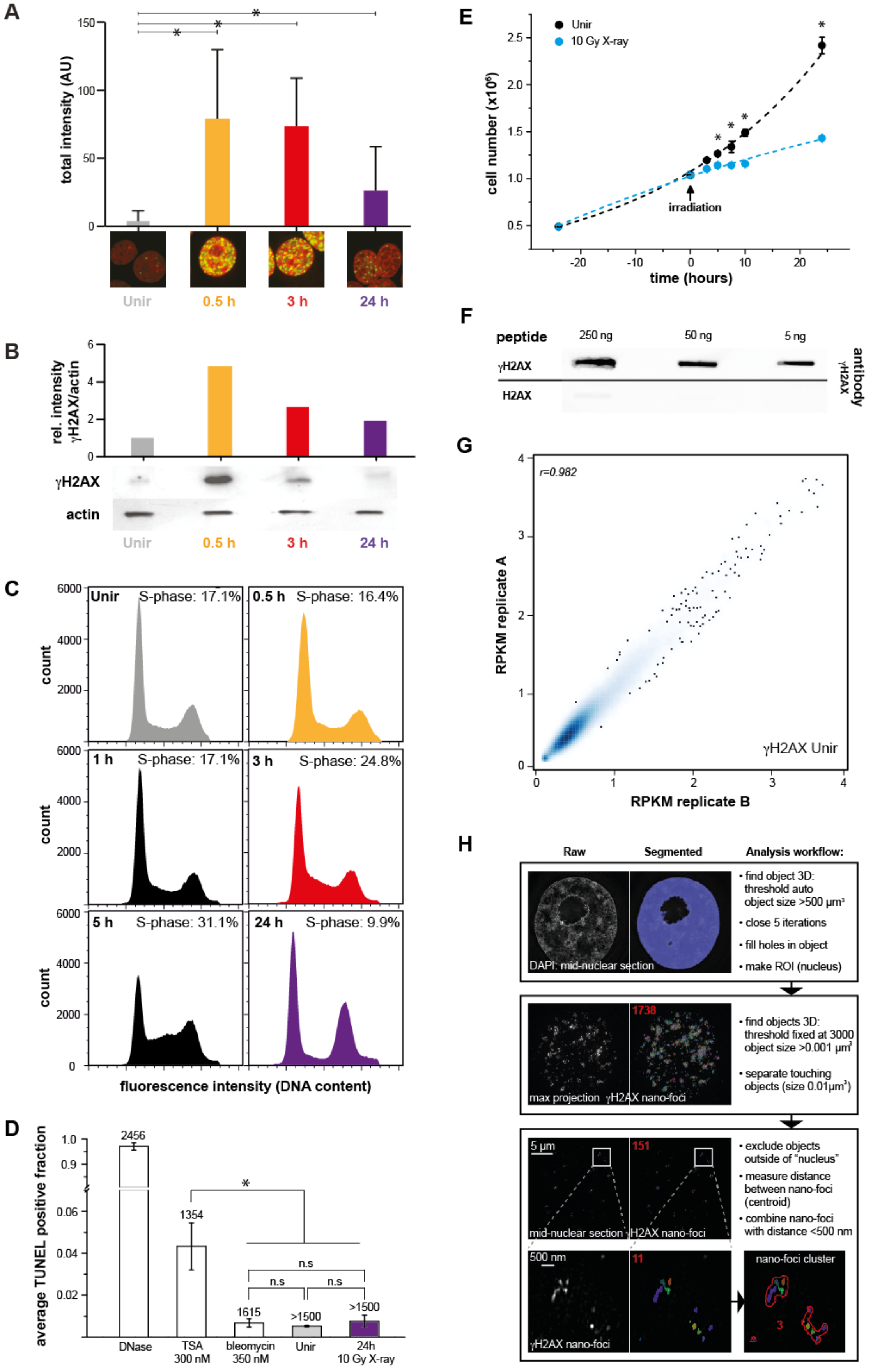
A
total intensity (AU)
Unir
0.5 h
3 h
24 h
B
rel. intensity γH2AX/actin
γH2AX
actin
Unir
0.5 h
3 h
24 h
C
Unir S-phase: 17.1%
0.5 h S-phase: 16.4%
1 h S-phase: 17.1%
3 h S-phase: 24.8%
5 h S-phase: 31.1%
24 h S-phase: 9.9%
count
fluorescence intensity (DNA content)
D
average TUNEL positive fraction
2456
1354
1615
>1500
>1500
n.s
DNase
TSA 300 nM
bleomycin 350 nM
Unir
24h 10 Gy X-ray
E
Unir
10 Gy X-ray
cell number (x10⁶)
irradiation
time (hours)
F
peptide
250 ng
50 ng
5 ng
γH2AX
H2AX
antibody γH2AX
G
r=0.982
RPKM replicate A
RPKM replicate B
γH2AX Unir
H
Raw
Segmented
Analysis workflow:
• find object 3D: threshold auto object size >500 µm³
• close 5 iterations
• fill holes in object
• make ROI (nucleus)
DAPI: mid-nuclear section
1738
• find objects 3D: threshold fixed at 3000 object size >0.001 µm³
• separate touching objects (size 0.01µm³)
max projection γH2AX nano-foci
5 µm
151
• exclude objects outside of "nucleus"
• measure distance between nano-foci (centroid)
• combine nano-foci with distance <500 nm
mid-nuclear section
H2AX nano-foci
500 nm
11
nano-foci cluster
γH2AX nano-foci

Supplementary Figure 1. Characterization and validation of cellular system and experimental strategy. To characterize the DDR as assessed by γH2AX formation, cells were exposed to 10 Gy X-ray and incubated as indicated. **(A)** Confocal microscopy immunofluorescence analysis of γH2AX before and after exposure to ionizing radiation (IR). Total γH2AX fluorescence intensity (Arbitrary Units) with exemplary micrographs matched to the corresponding time point is shown. In the micrograph: γH2AX (green); propidium iodide counterstained DNA (red). Results are mean and standard deviation from two independent experiments. *: significantly different from the mean of control unirradiated cells (one-way ANOVA, $p<10^{-4}$). **(B)** Immunoblot analysis of γH2AX (top blot) before and after exposure to IR. Loading control: β-actin (bottom blot). The ratios between γH2AX and β-actin chemiluminescence signal intensities is normalized to one for the unirradiated sample (Unir) and shown as bars in an exemplary barplot. **(C)** Cell cycle analysis by flow cytometry. After exposure to IR, cells underwent cell cycle arrest and accumulated in S-phase up to five hours post IR. After repair of DNA damage, cells progressed from the S-phase arrest into G2-phase (24h). Note the reduced population in S-phase 24h post IR. Fraction of cells in S-phase is indicated in each box. Two independent experiments were performed and ~25,000 cells per time point were analyzed. **(D)** Apoptosis analysis by TUNEL assay. Cells were treated with TSA or bleomycin at the indicated concentrations, or exposed to 10 Gy X-ray and incubated for 24h prior to analysis. “DNAse” (positive control) and unirradiated sample (negative control) are included. Results are mean and standard deviation from two independent experiments (n = 30; 15 imaged fields per condition per experiment). Total numbers of screened cells for each sample are indicated above each bar. The fraction of apoptotic cells never exceeded 1% in irradiated cells. *: two-tailed t-test, $p<10^{-4}$. n.s.: not significant. **(E)** Growth curve of cells before and after IR (cyan curve) as opposed to unirradiated control (black curve). Cells were seeded 24h before irradiation or mock-irradiation and cell number was assessed at indicated times. Note that after growth arrest, cells re-entered cell cycle and started proliferating again (24h post IR). Results

represent mean ± SEM from three independent growth curves, each performed in triplicate. *: two-tailed t-test, $p<10^{-2}$. **(F)** Slot blot analysis to test γH2AX antibody specificity. The γH2AX and H2AX peptides used for immunization were blotted at increasing indicated amount. The membrane was then probed with γH2AX antibody. Little (250 ng) to no cross-reactivity of anti-γH2AX antibody with H2AX peptide was observed. **(G)** ChIP-Seq reproducibility was assessed by comparing the RPKM values from two biological replicates. γH2AX ChIP and ChIP-Seq library preparation from two independent experiments are compared. γH2AX RPKM values were computed in 10 kbp genomic intervals, totalling 286,729 intervals. The two biological replicates show high linear correlation, with a Pearson's r of 0.982 ($p<2.2\times10^{-16}$). **(H)** Workflow of the image analysis protocol to quantify 3D-SIM data, including nuclear segmentation (top), γH2AX (nano-)foci segmentation (mid) and cluster analysis (bottom). A minimum segmentation unit of 2×2×2 voxels was allowed. An exemplary cell from the 24h time point is shown, together with the number of foci/clusters at all stages (in red). A detailed protocol of the microscopy analysis is in the "Image analysis" section in Methods.

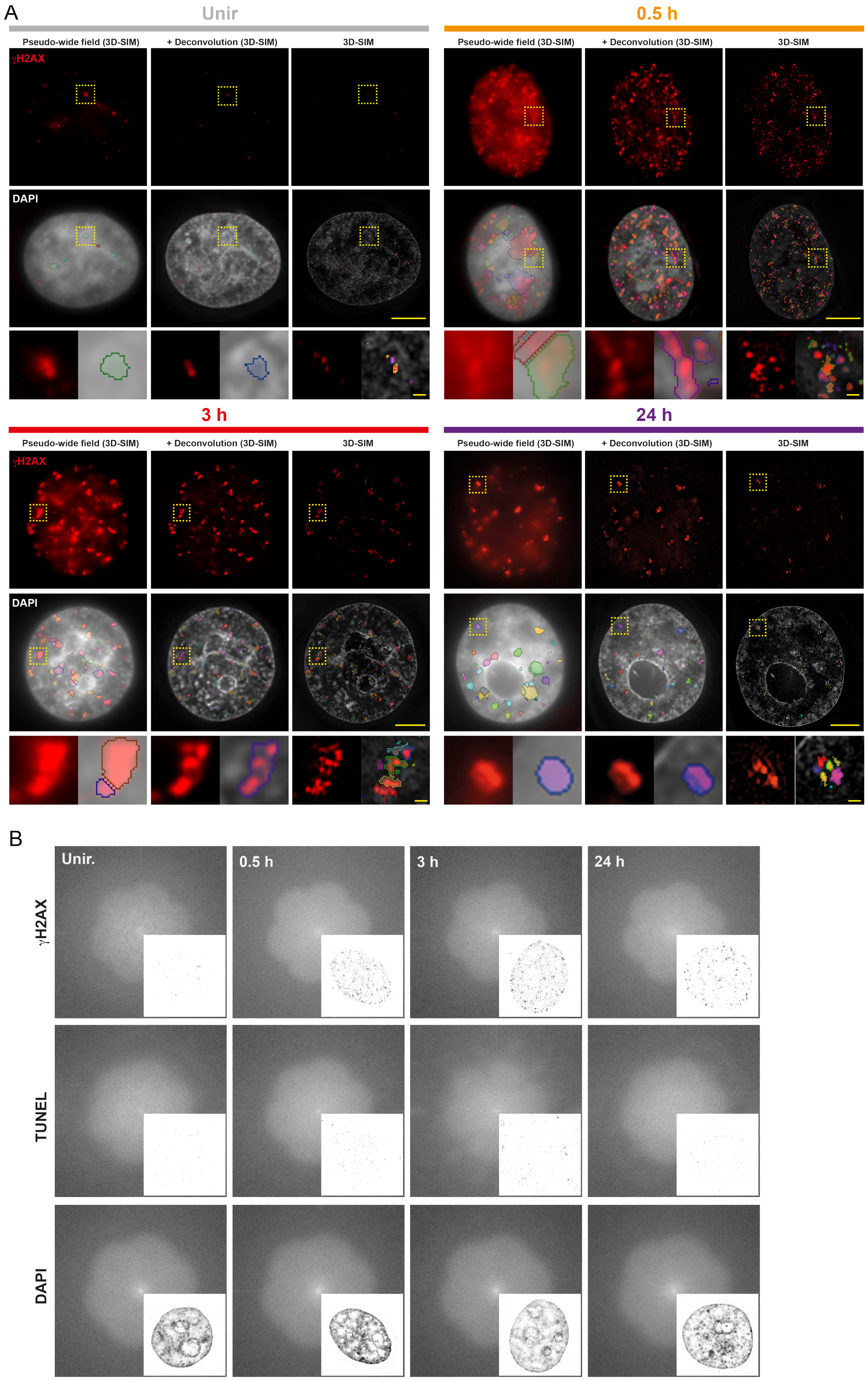
A
Unir
0.5 h
Pseudo-wide field (3D-SIM)
+ Deconvolution (3D-SIM)
3D-SIM
γH2AX
DAPI
3 h
24 h
B
Unir.
0.5 h
3 h
24 h
γH2AX
TUNEL
DAPI

Supplementary Figure 2. (A) Segmentation of γH2AX nano-foci in 3D-SIM images before and during the DNA damage response. Mid-nuclear section (*z*: 125 nm) of representative images of cells before or after exposure to 10 Gy X-ray. The same cell is shown as re-computed pseudo-wide field image before or after deconvolution, as well as the original 3D-SIM output. The total number of segmented focal structures is presented in the middle panel, together with DAPI. The lower panels show magnified views of the yellow dashed frame. Scale bars: 5 µm and 500 nm for main micrographs and magnified regions, respectively. **(B)** 3D-SIM images represented in the Fourier's space. To avoid reconstruction artifacts, the images were controlled in Fourier space. Here, sample images from the γH2AX and TUNEL co-staining are presented with the Fast Fourier Transformed (FFT) images of mid nuclear sections together with the underlying images as insets. No reconstruction artifacts are visible in the information containing central rosettes.

A

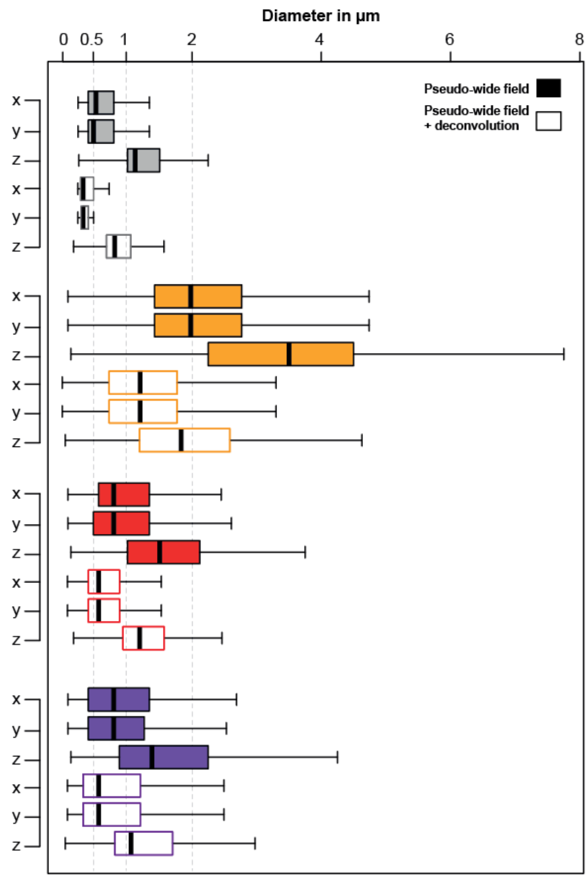

B

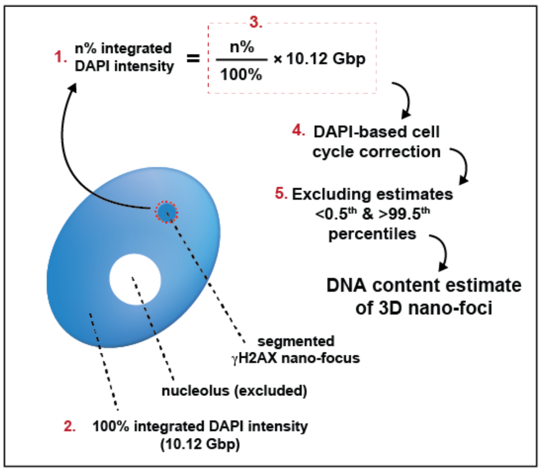

C

SKY Results: Pseudo-chromosome length estimate

Chr	Frequency (haploid)	Human chromosome length (bp)	Frequency adjusted chromosome length (haploid; bp)
1	1.95	248,956,422	486,596,643
2	1.50	242,193,529	363,290,293
3	1.05	198,295,559	207,308,984
4	1.05	190,214,555	198,860,662
5	3.36	181,538,259	610,628,714
6	1.05	170,805,979	178,569,879
7	2.00	159,345,973	318,691,946
8	2.09	145,138,636	303,471,680
9	1.50	138,394,717	207,592,075
10	1.45	133,797,422	194,614,438
11	1.50	135,086,622	202,629,933
12	2.00	133,275,309	266,550,618
13	1.00	114,364,328	114,364,328
14	1.50	107,043,718	160,565,577
15	1.91	101,991,189	194,710,461
16	1.50	90,338,345	135,507,517
17	2.00	83,257,441	166,514,882
18	1.50	80,373,285	120,559,927
19	1.50	58,617,616	87,926,424
20	1.82	64,444,167	117,171,224
21	1.45	46,709,983	67,941,795
22	1.00	50,818,468	50,818,468
X	1.95	156,040,895	304,989,029
Y	0.00	57,227,415	0
			Sum: 5,059,875,502

Summary Karyotype

N	46
Mean	73.9
Median	74
SD	2.678
SEM	0.395
Lower 95% CI of Mean	73.03
Upper 95% CI of Mean	74.62
Minimum	61
Maximum	81

D

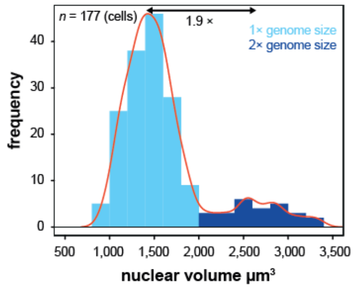

Supplementary Figure 3. Spectral karyotyping analysis of HeLa cells and DAPI-based cell cycle correction. **(A)** Quantification of the lateral and axial diameters of segmented objects in re-computed images, before and during DDR. The difference between lateral and axial measurements is due to the decreased resolution in the axial direction. **(B)** Schematic representation of the measurement of γH2AX nano-foci DNA content. The

whole procedure is summarized in bullet points as follows: i) the nucleus of a cell (excluding the nucleoli) and each γH2AX nano-focus are segmented; ii) the sum of all voxel in the segmented nucleus corresponds to the total integrated DAPI intensity (indicated as „2.“); iii) for each γH2AX nano-focus, the DAPI values of each voxel belonging to the segmented volume are summed (indicated as „1.“); iv) the resulting values are then normalized over the total integrated DAPI intensity (indicated as „3.“); v) this provides the fraction of total DAPI embedded in a single nano-focus, independent of the local DNA condensation state; vi) finally, to estimate the DNA content, the DNA fractions were corrected for the total genome size (determined by spectral karyotyping, panel C) and the cell cycle phase (panel D) **(C)** Relative haploid chromosome frequencies were combined with the human reference chromosome length to generate frequency-adjusted haploid pseudo-chromosomes. The total pseudo-haploid genome (5.06 Gbp) is the sum of all pseudo-chromosomes. A summary of all statistics from SKY is shown in the bottom box and reveals HeLa quasi-tetraploidy. **(D)** Distribution of the nuclear volume of all wild type cells analyzed during the DDR (n = 177). The distribution was arbitrarily split into two halves, and the corresponding “genome size” correction factor was used to adjust the nano-foci size (Fig. 2C). The major contribution to the nano-foci size is provided by the “1× genome” fraction.

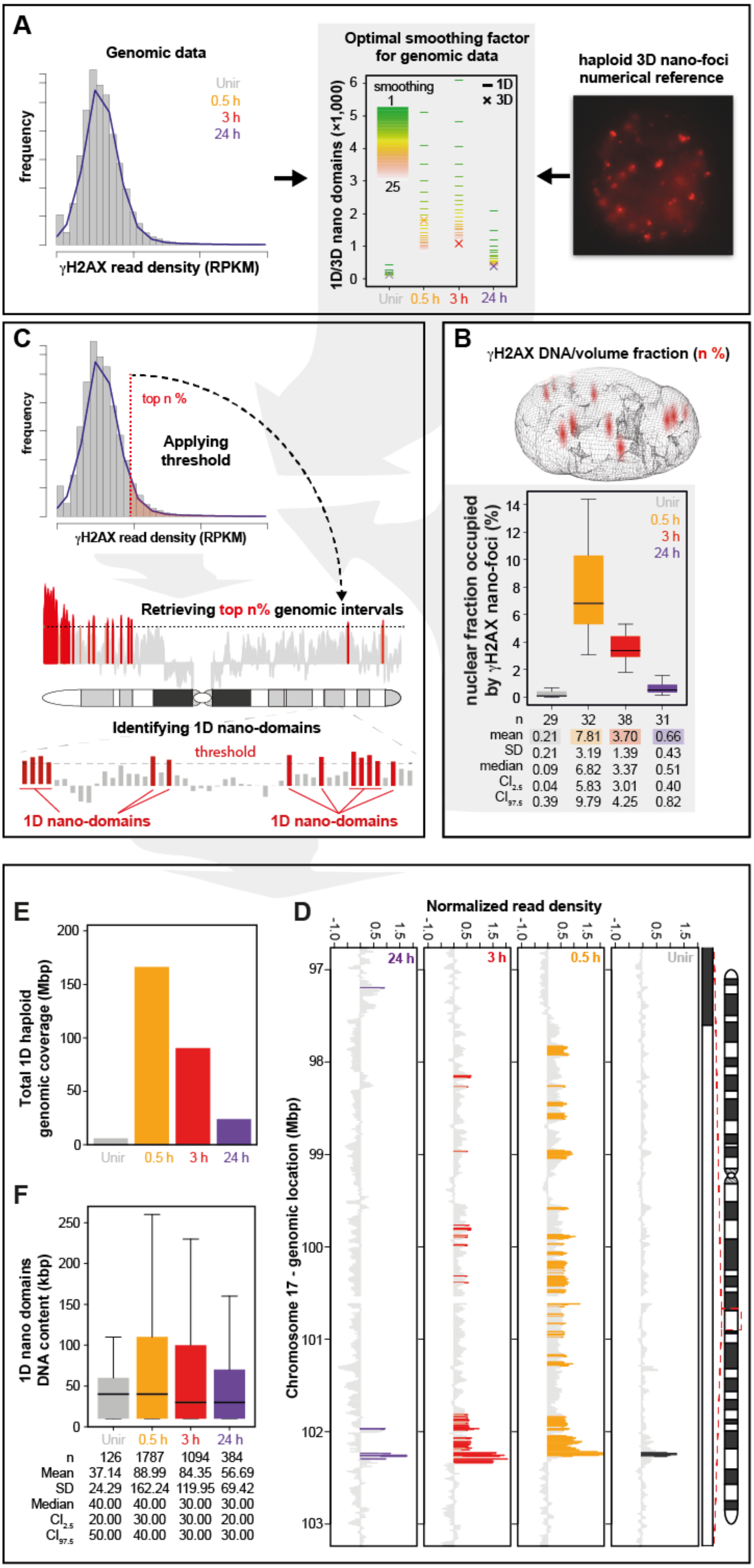
A
Genomic data
frequency
γH2AX read density (RPKM)
Unir
0.5 h
3 h
24 h
Optimal smoothing factor
for genomic data
1D/3D nano domains (×1,000)
smoothing
1
25
1D
× 3D
haploid 3D nano-foci
numerical reference
C
top n %
Applying
threshold
Retrieving top n% genomic intervals
Identifying 1D nano-domains
threshold
1D nano-domains
1D nano-domains
B
γH2AX DNA/volume fraction (n %)
nuclear fraction occupied
by γH2AX nano-foci (%)
n 29 32 38 31
mean 0.21 7.81 3.70 0.66
SD 0.21 3.19 1.39 0.43
median 0.09 6.82 3.37 0.51
CI2.5 0.04 5.83 3.01 0.40
CI97.5 0.39 9.79 4.25 0.82
E
Total 1D haploid
genomic coverage (Mbp)
F
1D nano domains
DNA content (kbp)
n 126 1787 1094 384
Mean 37.14 88.99 84.35 56.69
SD 24.29 162.24 119.95 69.42
Median 40.00 40.00 30.00 30.00
CI2.5 20.00 30.00 30.00 20.00
CI97.5 50.00 40.00 30.00 30.00
D
Normalized read density
Chromosome 17 - genomic location (Mbp)

Supplementary Figure 4. Integration of 3D-SIM and ChIP-Seq data to estimate the size of genomic γH2AX-decorated chromatin. (**A**) generation of 25 independent profiles by applying a smoothing factor (moving average) to each γH2AX ChIP-Seq dataset (middle panel, coloured lines). Such smoothing factor is a moving average ranging from 1 (no smoothing) to 25 genomic intervals (indicated as “1D”). Crosses (indicated as “3D”) are the ploidy-corrected 3D-SIM γH2AX nano-foci. The smoothing factor is chosen according to the best fit between genomic and microscopy data. (**B**) Volume fraction occupied by γH2AX nano-foci as well as their corresponding DNA content, before and during the DNA damage response. (**C**) Filtering the previously smoothed genomic γH2AX ChIP-Seq data by applying the mean volume fractions measured in (B), so that only the 10 kbp genomic intervals from the top-percentiles of the read density distributions at matched time-points were retrieved. (**D**) Exemplary panel showing the filtered intervals from the underlying ChIP-Seq profiles. (**E**) Linear coverage of the filtered ChIP-Seq datasets. The total genomic coverage corresponds to the DNA content estimate we measured in the mean volume fraction from (B). (**F**) Estimate of the 1D domain size distribution.

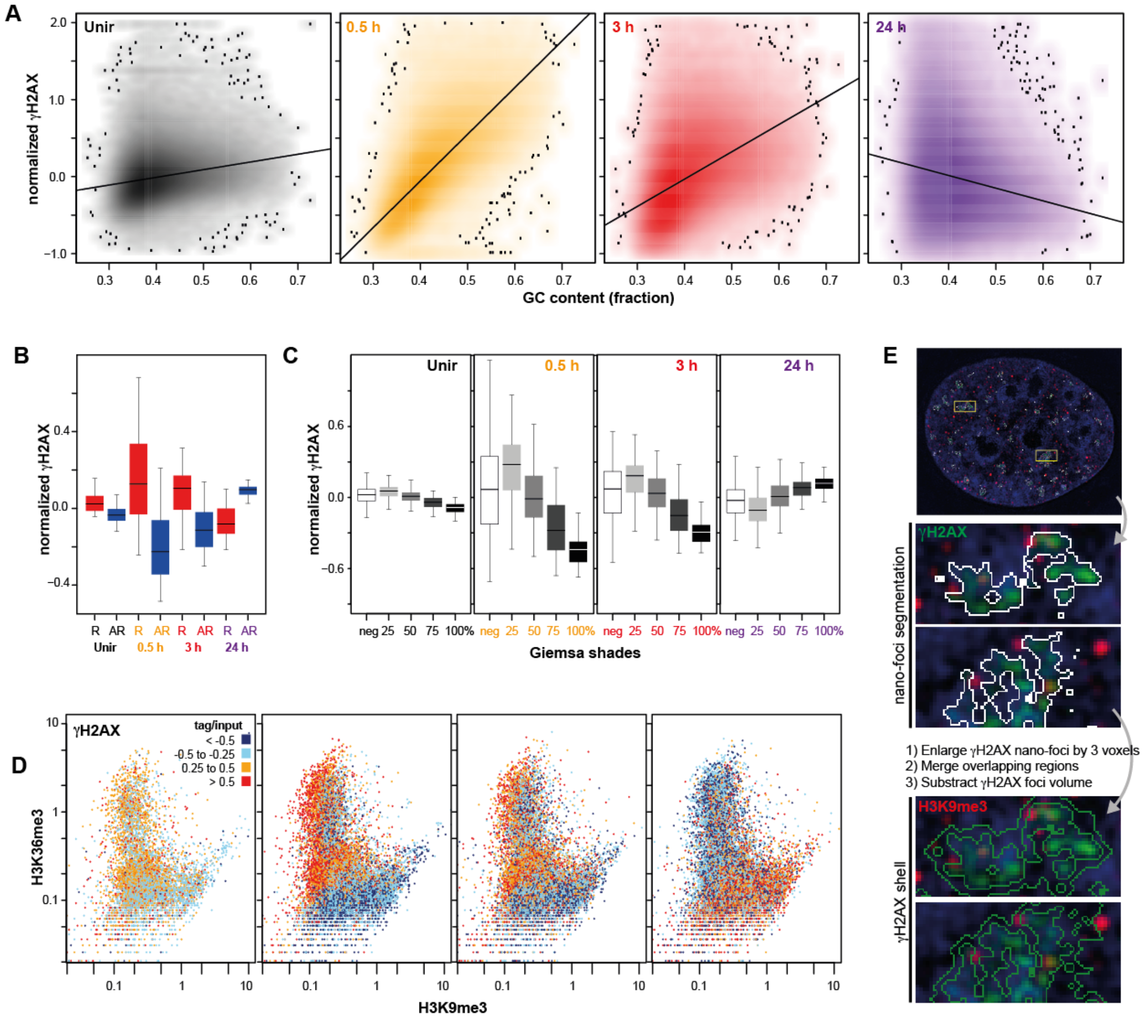

Supplementary Figure 5. Genomic and microscopic analysis of γH2AX-decorated chromatin. **(A)** Genome-wide correlation between γH2AX levels and GC content before and after IR. Normalized levels were calculated as follows: [(γH2AX$_{interval}$ RPKM/input$_{interval}$ RPKM)-(γH2AX$_{average}$ RPKM/input$_{average}$ RPKM)], where “interval” is a 10 kbp genomic interval and “average” is the genome-wide RPKM average value of all intervals in each corresponding dataset. Data are presented as density scatter plots of normalized γH2AX levels as a function of GC content. The early (0.5h, orange) mid- (3h, red) and late (24h, purple) stages of DDR as well as the sham-irradiated levels (Unir, grey) are shown. Black line: linear regression. Positive correlation with increasing GC content was observed before and up to 3h post IR. At 24h, the tendency was inverted, as indicated by the negative slope of the regression line. **(B)** γH2AX levels in

(anti-)RIDGEs before and after IR. Normalized levels are presented as [(γH2AX $_{(anti-)RIDGE}$ RPKM/input$_{(anti-)RIDGE}$ RPKM) - (γH2AX$_{average}$ RPKM/input$_{average}$ RPKM)] where "(anti-)RIDGE" is the total genomic coverage for all RIDGEs or anti-RIDGEs and "average" is the genome-wide RPKM average value of all genomic intervals. Upon IR, γH2AX is enriched in RIDGEs, whereas at later times the trend is inverted. Wilcoxon rank sum rest; $p<10^{-5}$. **(C)** γH2AX levels in Giemsa-shaded band ideograms before and after IR. Normalized levels are presented as [(γH2AX$_{band\ type}$ RPKM/input$_{band\ type}$ RPKM) - (γH2AX$_{average}$ RPKM/input$_{average}$ RPKM)] where "band type" is the total genomic coverage for each band and "average" is the genome-wide RPKM average value of all genomic intervals. Upon IR, γH2AX is enriched in Giemsa light bands (negative and 25%) whereas at later times the trend is inverted (75-100%). Kruskal-Wallis test and p-values in Supplementary Table 4. **(D)** Genome-wide γH2AX levels before and after IR. Each dot in the scatterplot represents a 10 kbp genomic interval whose coordinates correspond to H3K9me3 (x-axis) and H3K36me3 (y-axis) levels. The relative γH2AX enrichment in each genomic interval is presented as a heat-map, increasing from blue to red. It is to be noted that, upon IR, γH2AX is enriched in H3K36me3-rich/H3K9me3-poor compartments. Conversely, at later times, residual γH2AX signal is mainly found in H3K36me3-poor/H3K9me3-rich compartments. **(E)** γH2AX nano-foci (green) are segmented as described in Methods. The resulting volume units are then enlarged by three voxels in the three dimensions. All overlapping regions are merged to form a distinct volume unit. Finally, the volume of the original γH2AX nano-foci is subtracted to generate γH2AX shells. Fluorescence intensity of other probed features (e.g. H3K9me3, red) are then measured in the shells. The enlarged panels correspond to regions defined by the yellow frames. All boxes and whiskers represent 25-75 percentiles and three times the interquartile distance.

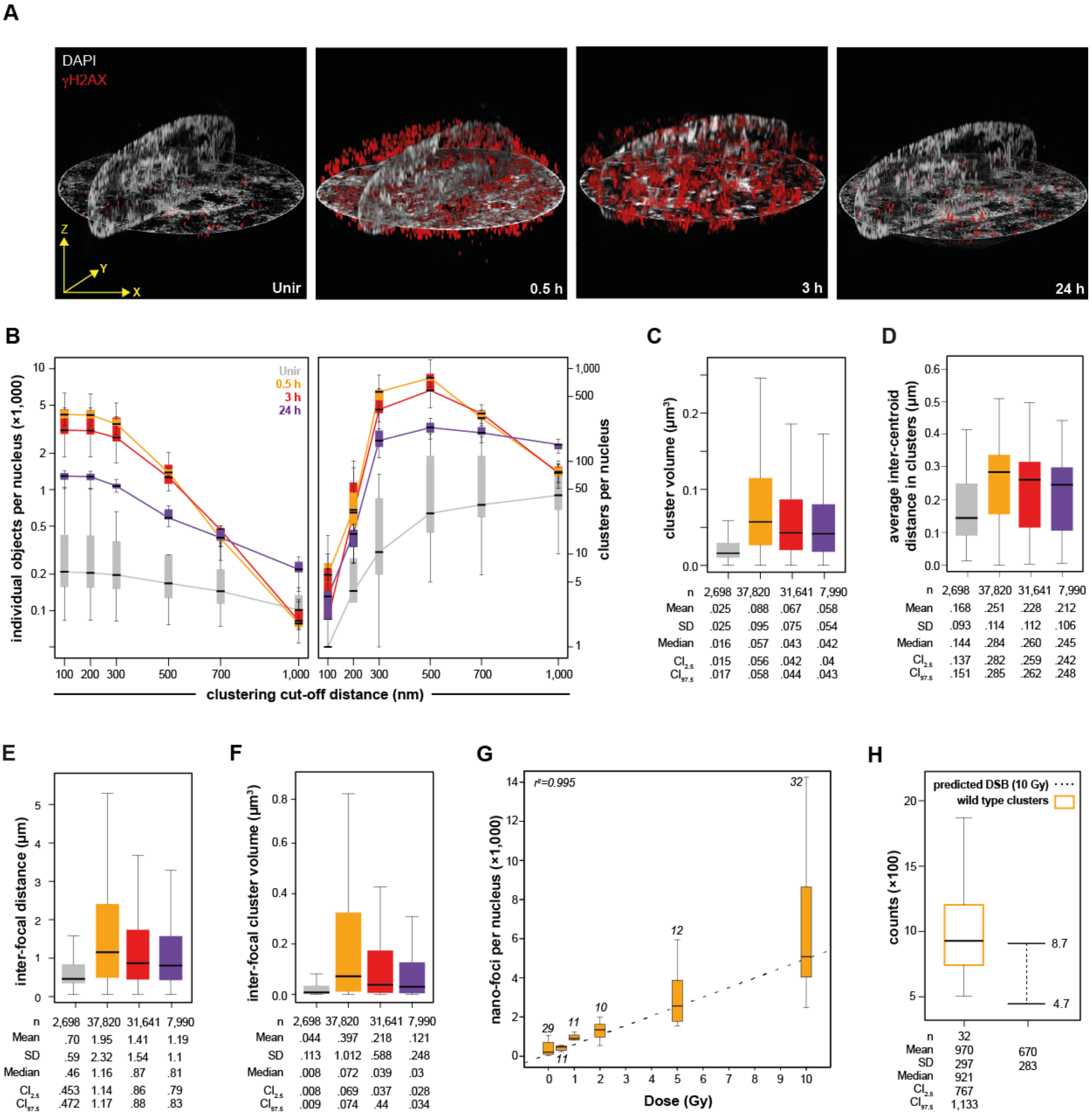

Supplementary Figure 6. Validation of γH2AX nano-foci and nano-foci clusters in cells. **(A)** Exemplary 3D-SIM images of γH2AX (red) before and during DDR showing a 3D representation of γH2AX nano-foci with DAPI channels in *xy* and *yz* mid-nuclear cross-sections. **(B)** Effect of cut-off distance between nano-foci for the cluster analysis. 10 cells per time point were analyzed for the effect of the clustering threshold distance (from 100 to 1,000 nm) and the resulting distributions are presented as boxplots. (left) Sum of 3D-clusters plus individual non-clustered nano-foci. (right) Total number of 3D-clusters. 500 nm was the cut-off distance resulting in both the highest number of clusters and clear repair kinetics. Solid lines connect the medians of each distribution.

(C) γH2AX 3D cluster integrated volume distributions. The volume of each nano-focus included in a cluster is summed. Kruskal-Wallis chi-squared = 2,941.4, df = 3, $p<2.2\times10^{-16}$. **(D)** Distribution of the average inter-centroid distances measured between each nano-focus belonging to a given cluster. Kruskal-Wallis chi-squared = 1,889.3, df = 3, $p<2.2\times10^{-16}$. **(E)** Distributions of the shortest paths connecting the centroids of all nano-foci belonging to a 3D cluster Kruskal-Wallis chi-squared = 2,223.7, df = 3, $p<2.2\times10^{-16}$. **(F)** Inter-focal 3D-clusters volume distributions, presented as the volume delimited by the centroids of each nano-focus belonging to a 3D-clusters Kruskal-Wallis chi-squared=2,217.5, df=3, $p<2.2\times10^{-16}$. **(G)** Dose-curve showing linear increase of γH2AX nano-foci. Cells were irradiated with 0.5, 1, 2, 5 and 10 Gy X-ray and incubated 0.5h before fixation. γH2AX immunofluorescence was followed by nano-foci quantification on 3D-SIM images. The number of imaged cells per dose is shown in italic. Dashed line: linear regression calculated over the median of each distribution, after subtracting the median number of nano-foci from unirradiated cells. Estimated nano-foci per Gy: 495, after background subtraction. Kruskal-Wallis chi-squared = 88.028, df = 5, $p<2.2\times10^{-16}$. All boxes and whiskers are 25th-75th percentile and three times the interquartile distance, respectively. n: number of analyzed 3D clusters. **(H)** Comparison between the numbers of γH2AX clusters, 0.5h post IR and the predicted number of DSBs induced by 10 Gy X-ray.

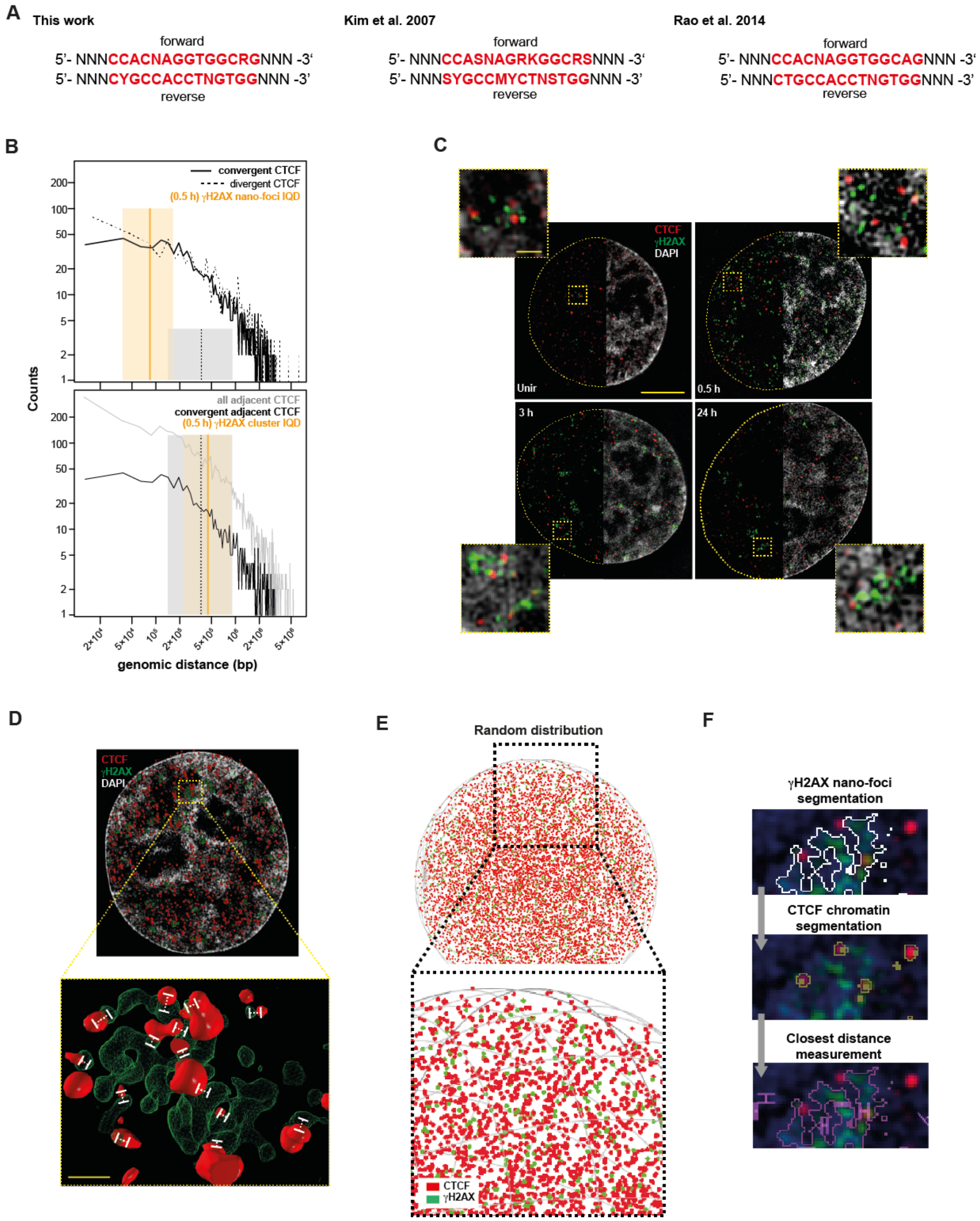

Supplementary Figure 7. Spatial localization of CTCF with respect of γH2AX-decorated chromatin. (A) CTCF consensus motifs used in this work and from previous works. **(B)** Size comparison between CTCF-delimited chromatin segments and γH2AX 3D-nano-foci and clusters. Because the CTCF motif is not a palindrome, two adjacent motifs can have four possible orientations. Recent findings indicate that, when a

chromatin loop is formed, the CTCF sites are facing one another in a convergent fashion in almost all cases. The distances between all adjacent CTCF genomic sites independent of their orientation (grey curve) as well as those between two adjacent convergent (black solid curves) or divergent (dashed curves) CTCF genomic sites are shown. The IQD of the “convergent adjacent” distribution (grey box; dashed line: median) is compared to that of γH2AX 3D clusters (left; orange box; line: median) or γH2AX nano-foci size (right; orange box; line: median). Little to no difference is observed when comparing distances between convergent adjacent CTCF sites and distances between random or divergent orientation (IQD: 150-987 kbp). **(C)** 3D-SIM images of immuno-stained γH2AX and CTCF before and during DDR. The DAPI channel represents the mid-nuclear section. The dotted curved line delimits the nuclear contour. Panels on the right are enlarged views of a representative region (yellow dashed lines). **(D)** Three-dimensional rendering of γH2AX (green) and CTCF (red) immunostaining in a mid nuclear section, 24h after IR. The enlarged region represents γH2AX foci clusters surrounded by CTCF. White dashed lines: exemplary measurements. **(E)** Graphical representation of simulated γH2AX and CTCF foci 0.5h post IR in a sphere of volume comparable to that of a cell nucleus. The number of γH2AX and CTCF foci used in the simulation are matched to the number of foci detected in 3D-SIM images of each time-point. Specifically, 5,348, 6,731, 8,154, 7,497 CTCF and 374, 4,357, 4,065 and 1,200 γH2AX nano-foci were used for unirradiated, 0.5h, 3h and 24h time points, respectively. **(F)** Shell segmentation and analysis workflow for the measurement of CTCF proximity to γH2AX foci: i) γH2AX foci are segmented; ii) CTCF foci are segmented; iii) the closest Euclidian distance between the centroids of γH2AX and CTCF foci is measured.

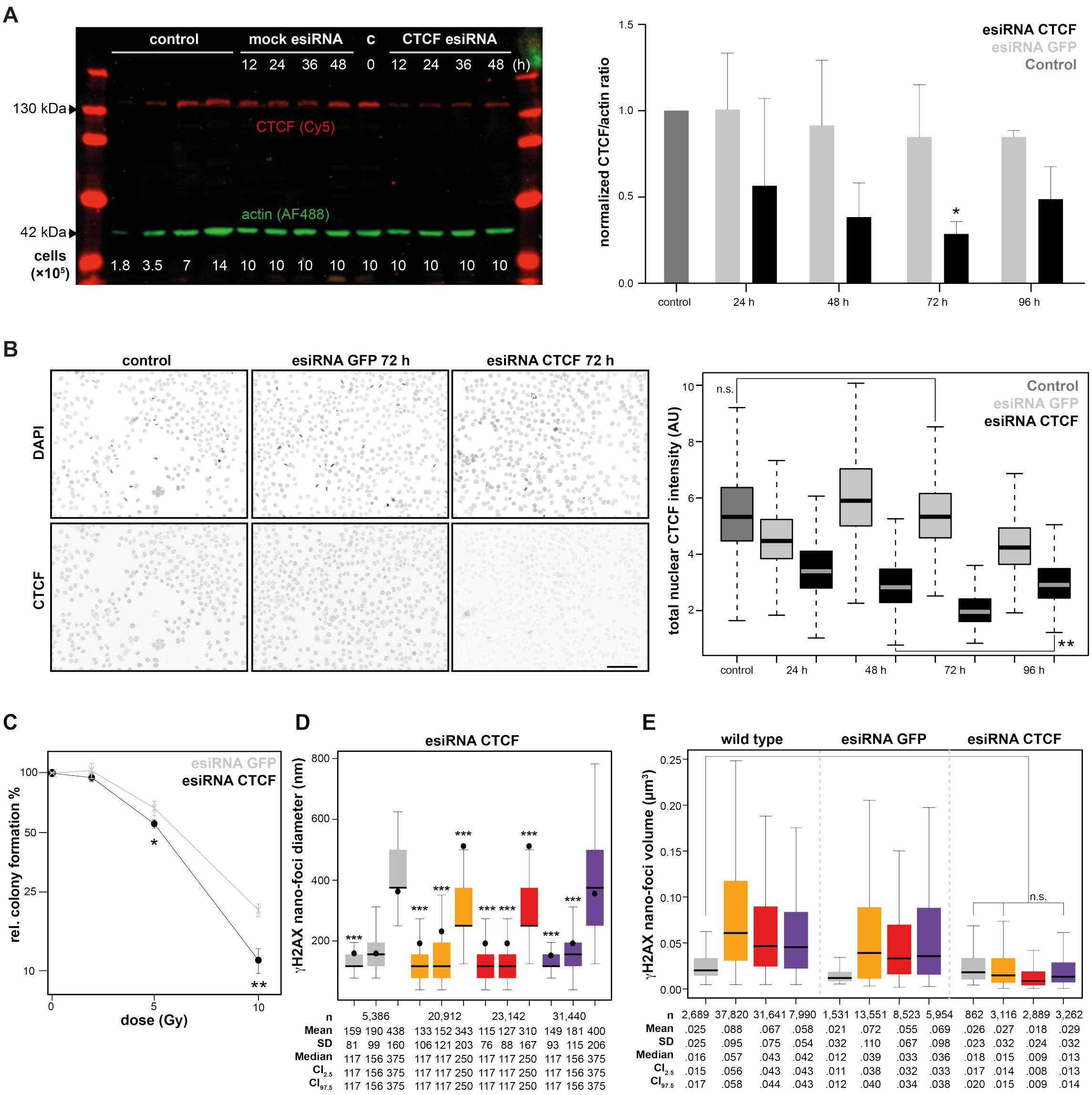

Supplementary Figure 8. CTCF knock-down via RNAi. (A) Left, representative immunoblot of CTCF protein in the absence or presence of CTCF-esiRNA; lanes 1 and 15: protein ladder; lanes 2 to 5: loading control with increasing amount from left to right (the cell number is indicated below); lanes 6 to 9: mock (GFP) esiRNA, quadruplicate; lane 10: untransfected control; lanes 11 to 14: CTCF esiRNA, quadruplicate. Right, quantification of CTCF protein levels, relative to the untransfected control. 72h after incubation with CTCF esiRNA, we observed the maximum depletion, with CTCF protein levels being about 40%, compared to the corresponding mock-treated sample (ANOVA with Dunnett's correction; *: $p<0.05$, relative to control). **(B)** High-content immunofluorescence microscopy of cells in which CTCF protein was knocked-down via

esiRNA. Wide field images of DNA (up) and CTCF (down) immunofluorescence in the absence or presence of CTCF-esiRNA for the indicated times are shown. GFP esiRNA (middle panels) was used as mock transfection control. Scale bar: 100 µm. Right, the boxes are the distributions of total nuclear CTCF fluorescence intensity from at least 4,500 cells at the indicated times post esiRNA treatment. Kruskal-Wallis test with Dunn's multiple comparison correction. **: $p<10^{-2}$; n.s.: not significant; all other pairs are significantly different with a $p<10^{-3}$. **(C)** Diminished CTCF levels increase radiosensitivity. Colony formation assay was performed after exposing cells to the indicated X-ray doses. Values are mean and standard deviation from two independent assays. For each experiment, 3 and 6 technical replicates, for unirradiated and irradiated samples at the indicated doses, respectively, were analyzed. Two-tailed t-test with $p<0.05$ (*) or 0.01 (**). **(D)** Quantification of γH2AX nano-foci diameters in CTCF siRNA-treated cells before and after IR. Black dots: median length of γH2AX nano-foci diameters in untreated cells (from Fig. 2A). ***: Wilcoxon rank-sum test, $p<<10^{-3}$. **(E)** γH2AX 3D-clusters integrated volume distributions (as in Supplementary Fig. 6C) in untreated (control), mock- and CTCF-depleted cells. n.s.: Kruskal-Wallis test with Dunn's multiple comparison, with $p>0.05$.

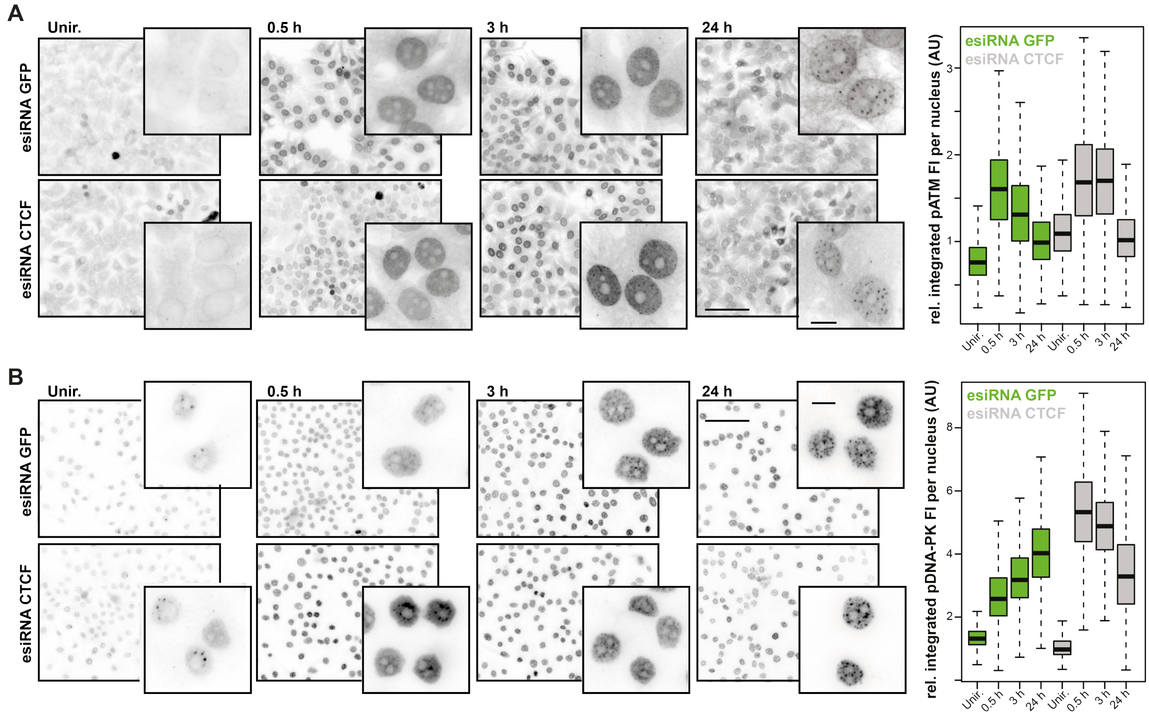

Supplementary Figure 9. High content immunofluorescence microscopy of phospho-ATM **(A)** or phospho-DNA-PKcs **(B)** before or during DDR, in CTCF-depleted cells. Briefly, cells were exposed to 10 Gy IR, incubated for the indicated times and then fixed. High content immunofluorescence microscopy and analysis were then performed with an Operetta System. Scale bar: 100 µm, inset scale bar: 10 µm. Results are from two independent experiments, with >5,000 individual cells per condition per time-point analyzed. Kruskal-Wallis test with Dunn's multiple comparison correction; all pairs are significantly different with a $p<10^{-3}$. All boxes and whiskers represent 25-75 percentiles and three times the interquartile distance.

Supplementary Table 1

DNA content of γH2AX nano-foci and clusters

3D nano-foci (kbp)	Min.	LowQ	Med.	Mean	SD	UpQ	Max
Unirradiated (*n* = 16,798)	0.8	23.8	38.9	53.6	48.1	64.9	369.2
0.5 h (*n* = 233,515)	0.7	31.7	69.7	119.9	142.5	148.5	1,100.0
3 h (*n* = 166,841)	1.9	27.9	57.8	106.6	138.3	125.9	1,281.0
24 h (*n* = 50,143)	0.7	17.8	32.7	80.8	174.7	67.7	2,008.0
**3D clusters (kbp)**	**Min.**	**LowQ**	**Med.**	**Mean**	**SD**	**UpQ**	**Max**
Unirradiated (*n* = 2,698)	21.1	81.7	132.1	190.7	173.8	234.6	1,624
0.5 h (*n* = 37,820)	20.7	195.3	424.2	687.2	782.6	884.6	10,224
3 h (*n* = 31,641)	21.7	141.5	300.7	478.9	547.8	622.1	12,251
24 h (*n* = 7,990)	20.9	87.7	189.1	286.9	299.8	377.3	3,746

Supplementary Table 2

Ploidy-corrected DNA content of γH2AX nano-foci and clusters

3D nano-foci (kbp) ploidy-corrected	Min.	LowQ	Med.	Mean	SD	UpQ	Max
Unirradiated (*n* = 16,798)	0.7	23.4	38.9	53.2	48.1	64.9	369.2
0.5 h (*n* = 233,515)	0.7	33.7	74.6	126.9	149.9	159.1	1,137.0
3 h (*n* = 166,841)	1.9	27.9	57.4	106.2	138.2	125.2	1,281.0
24 h (*n* = 50,143)	0.7	25.1	48.1	104.2	180.2	104.4	2,008.0
**3D clusters (kbp)**	**Min.**	**LowQ**	**Med.**	**Mean**	**SD**	**UpQ**	**Max**
Unirradiated (*n* = 2,698)	2.1	74.2	123.3	178.1	154.2	223.1	1,035.8
0.5 h (*n* = 37,820)	10.2	197.3	440.8	710.2	767.3	937.7	5,324.3
3 h (*n* = 31,641)	15.1	136.8	296.1	469.8	469.7	622.4	3,539.2
24 h (*n* = 7,990)	4.3	111.9	269.2	400.3	389.8	553.9	2,439.3

n: number of nano-foci (top) or clusters (bottom); Min., Max.: minimum and maximum value in the distribution; LowQ, UpQ: 25th and 75th percentiles of the distribution; Med.: median; SD: standard deviation.

Supplementary Table 3

Overview of genomic features used

Feature	Cell Type	Type of Data	Data Source / Reference
**General Features**			
G-banding	Human	% Shading	UCSC Genome Brower
Distance to the telomere	Hg19	Distance in bp	UCSC Genome Brower
Distance to the centromere	Hg19	Distance in bp	UCSC Genome Brower
Purine percent	Hg19	Percentage	In-house calculation
GC content	Hg19	Percentage	In-house calculation
DNase	HepG2	DNase-seq	GSM816662
FAIRE	HepG2	FAIRE-seq	GSM864354
CpG island	Hg19	Count	UCSC Genome Brower
**Transcription**			
miRNA	Human	Count	miRBase[1]
TSS	Hg19	Distance in bp	UCSC Genome Brower
Expression	HepG2	Micro array	GSM646144-5[2]
Rel. Pol2	HepG2	Chip-Seq	GSM822284
Rel. Pol2_S2	HepG2	Chip-Seq	GSM935543
RIDGES	Human	Coordinates	http://r2.amc.nl
Genic region	Hg19	Count	UCSC Genome Brower
**DNA Methylation**			
Average DNA Methylation	HepG2	Micro array	GSM999338
Number of DNA methylation sites (No. DNA Methyl.)	HepG2	Micro array count	GSM999338
Relative MBD4 abundance (Rel. MBD4)	HepG2	ChIP-seq	GSM1010740
**Histones and Histone Modifications**			
H2A.Z	HepG2	Chip-Seq	GSM733774[3]
H3K4me1	HepG2	Chip-Seq	GSM798321[3]
H3K36me3	HepG2	Chip-Seq	GSM733685[3]
H3K9me3	HepG2	Chip-Seq	GSM1003519[3]
H3K79me2	HepG2	Chip-Seq	GSM733641[3]
H3K27ac	HepG2	Chip-Seq	[3]
H3K27me3	HepG2	Chip-Seq	[3]
H3K4me2	HepG2	Chip-Seq	[3]
H3K4me3	HepG2	Chip-Seq	[3]
H3K9ac	HepG2	Chip-Seq	[3]
H4K20me1	HepG2	Chip-Seq	[3]
**DNA Sequence Elements**			
Alu repeats	Human	Count	RepeatMasker[4]
MIR repeats	Human	Count	RepeatMasker[4]
LINE1 repeats	Human	Count	RepeatMasker[4]
LINE2 repeats	Human	Count	RepeatMasker[4]
MER repeats	Human	Count	RepeatMasker[4]
AT Low Complexity repeats	Human	Count	RepeatMasker[4]
GC Low Complexity repeats	Human	Count	RepeatMasker[4]
Simple repeats	Human	Count	RepeatMasker[4]
G-Quadruplex Forming repeats (Quadruplex repeats)	Human	Count	RepeatMasker[4]
Z-DNA Motif	Human	Count	[5]
Z-DNA hotspot	Human	Count	[5]
Inverted repeats	Human	Count	[5]
Cruciform Motif	Human	Count	[5]
Direct repeats	Human	Count	[5]
Slipped Motif	Human	Count	[5]

Mirror repeats	Human	Count	[5]
Triplex Motif	Human	Count	[5]
A-Phased repeats	Human	Count	[5]
Microsatellite	Human	Count	RepeatMasker[4]
**DNA Replication**			
Replication timing S1	GM12801	RepliSeq	GSM923440[6]
Replication timing S2	GM12801	RepliSeq	GSM923440[6]
Replication timing S3	GM12801	RepliSeq	GSM923440[6]
Replication timing S4	GM12801	RepliSeq	GSM923440[6]
Replication timing G1b	GM12801	RepliSeq	GSM923440[6]
Replication timing G2	GM12801	RepliSeq	GSM923440[6]
Origins of replication by lambda exonuclease digestion (Origin Replication Lexo)	HeLa	Genomic array	[7]
Origins of replication by anti-bromodeoxyuridine IP (Origin Replication BrIP)	HeLa	Genomic array	[7]
Origins of replication by common anti-bromodeoxyuridine IP and lambda exonuclease digestion (Lexo + BrIP)	HeLa	Genomic array	[7]
Origins of replication (Ori. Cadoret)	HeLa	Genomic array	[8]
Topoisomerase motif (Topo.CAT)	Hg19	Density	[9]
Topoisomerase motif (Topo.CTY)	Hg19	Density	[9]
Topoisomerase motif (Topo.GTY)	Hg19	Density	[9]
Topoisomerase motif (Topo.RAK)	Hg19	Density	[9]
Topoisomerase motif (Topo.YCCTT)	Hg19	Density	[9]
Topoisomerase motif (Topo.YTA)	Hg19	Density	[9]
**DNA Binding Factors**			
SMC3 (cohesin)	HepG2	ChIP-seq	GSM935542
Lamina Associated Domain	Tig3ET	Coverage	[10]
Rel.BRCA1	HepG2	ChIP-seq	[3]
Rel.Rad21	HepG2	ChIP-seq	[3]

Supplementary Table 4

Summary of statistical analyses related to figure S5

Figure	Sample	Test	p-value	Comment
**S5B**	Unir	Wilcoxon rank sum	2.91e-6	RIDGEs (xx) vs. anti-RIDGEs (xx)
**S5B**	0.5 h	Wilcoxon rank sum	2.98e-10	RIDGEs (xx) vs. anti-RIDGEs (xx)
**S5B**	3 h	Wilcoxon rank sum	2.95e-9	RIDGEs (xx) vs. anti-RIDGEs (xx)
**S5B**	24 h	Wilcoxon rank sum	< 2.2e-16	RIDGEs (xx) vs. anti-RIDGEs (xx)
**S5C**	Unir “0 vs 25”	Kruskal-Wallis	< 5e-2	Giemsa bands group comparison
**S5C**	Unir “0 vs 50”	Kruskal-Wallis	n.s.	
**S5C**	Unir “0 vs 75”	Kruskal-Wallis	< 1e-3	
**S5C**	Unir “0 vs 100”	Kruskal-Wallis	< 1e-3	
**S5C**	Unir “25 vs 50”	Kruskal-Wallis	< 1e-2	
**S5C**	Unir “25 vs 75”	Kruskal-Wallis	< 1e-3	
**S5C**	Unir “25 vs 100”	Kruskal-Wallis	< 1e-3	
**S5C**	Unir “50 vs 75”	Kruskal-Wallis	< 1e-3	
**S5C**	Unir “50 vs 100”	Kruskal-Wallis	< 1e-3	
**S5C**	Unir “75 vs 100”	Kruskal-Wallis	< 5e-2	
**S5C**	0.5 h “0 vs 25”	Kruskal-Wallis	< 1e-2	
**S5C**	0.5 h “0 vs 50”	Kruskal-Wallis	n.s.	
**S5C**	0.5 h “0 vs 75”	Kruskal-Wallis	< 1e-3	
**S5C**	0.5 h “0 vs 100”	Kruskal-Wallis	< 1e-3	
**S5C**	0.5 h “25 vs 50”	Kruskal-Wallis	< 1e-2	
**S5C**	0.5 h “25 vs 75”	Kruskal-Wallis	< 1e-3	
**S5C**	0.5 h “25 vs 100”	Kruskal-Wallis	< 1e-3	
**S5C**	0.5 h “50 vs 75”	Kruskal-Wallis	< 1e-3	
**S5C**	0.5 h “50 vs 100”	Kruskal-Wallis	< 1e-3	
**S5C**	0.5 h “75 vs 100”	Kruskal-Wallis	< 1e-2	
**S5C**	3 h “0 vs 25”	Kruskal-Wallis	< 1e-3	
**S5C**	3 h “0 vs 50”	Kruskal-Wallis	n.s.	
**S5C**	3 h “0 vs 75”	Kruskal-Wallis	< 1e-3	
**S5C**	3 h “0 vs 100”	Kruskal-Wallis	< 1e-3	
**S5C**	3 h “25 vs 50”	Kruskal-Wallis	< 1e-2	
**S5C**	3 h “25 vs 75”	Kruskal-Wallis	< 1e-3	
**S5C**	3 h “25 vs 100”	Kruskal-Wallis	< 1e-3	
**S5C**	3 h “50 vs 75”	Kruskal-Wallis	< 1e-3	
**S5C**	3 h “50 vs 100”	Kruskal-Wallis	< 1e-3	
**S5C**	3 h “75 vs 100”	Kruskal-Wallis	< 1e-2	
**S5C**	24 h “0 vs 25”	Kruskal-Wallis	< 1e-2	
**S5C**	24 h “0 vs 50”	Kruskal-Wallis	n.s.	
**S5C**	24 h “0 vs 75”	Kruskal-Wallis	< 1e-3	
**S5C**	24 h “0 vs 100”	Kruskal-Wallis	< 1e-3	
**S5C**	24 h “25 vs 50”	Kruskal-Wallis	< 1e-3	
**S5C**	24 h “25 vs 75”	Kruskal-Wallis	< 1e-3	
**S5C**	24 h “25 vs 100”	Kruskal-Wallis	< 1e-3	
**S5C**	24 h “50 vs 75”	Kruskal-Wallis	< 1e-2	
**S5C**	24 h “50 vs 100”	Kruskal-Wallis	< 1e-3	
**S5C**	24 h “75 vs 100”	Kruskal-Wallis	n.s.	

Supplementary Table 5

Summary of γH2AX (nano-)foci and cluster numbers

	γH2AX foci		γH2AX nano-foci							γH2AX nano-foci clusters		
	Confocal microscopy		pseudo-wide field		deconvolved pseudo-wide field		3D-SIM		% DDR*	3D-SIM cluster		% DDR*
Unir	13±10	(11)	46±33	(44)	75±26	(77.5)	392±347	(208)	(4.1)	68±70	(23)	(2.5)
0.5 h	53±20	(47)	268±56	(268)	427±83	(406)	6,287±2,785	(5,083.5)	(100)	970±297	(920.5)	(100)
3 h	44±14	(46)	194±73	(174)	361±111	(336)	3,603±1,148	(3,166.5)	(62.3)	663±171	(623)	(67.7)
24 h	23±19	(20)	128±55	(129)	197±45	(209)	1,210±406	(1,267)	(24.9)	203±74	(220)	(23.9)

Indicated are the mean number of γH2AX (nano-)foci ± SD as well as the median (in brackets).
*: assessed as percentage of γH2AX nano-foci or clusters relative to the median value from 0.5h (100%). Note that percentages are comparable between nano-foci and clusters, indicating that the cut-off distance from Supplementary Figure 6B did not impede the analysis of DDR.

Supplementary References

1. Griffiths-Jones, S., Saini, H.K., van Dongen, S. & Enright, A.J. miRBase: tools for microRNA genomics. *Nucleic Acids Res* **36**, D154-8 (2008).

2. Ernst, J. et al. Mapping and analysis of chromatin state dynamics in nine human cell types. *Nature* **473**, 43-9 (2011).

3. Encode Project Consortium. An integrated encyclopedia of DNA elements in the human genome. *Nature* **489**, 57-74 (2012).

4. Smit, A.F.A., Hubley, R. , Green, P. RepeatMasker Open 3.0. (1996-2010).

5. Cer, R.Z. et al. Non-B DB: a database of predicted non-B DNA-forming motifs in mammalian genomes. *Nucleic Acids Res* **39**, D383-91 (2011).

6. Hansen, R.S. et al. Sequencing newly replicated DNA reveals widespread plasticity in human replication timing. *Proc Natl Acad Sci U S A* **107**, 139-44 (2010).

7. Karnani, N., Taylor, C.M., Malhotra, A. & Dutta, A. Genomic study of replication initiation in human chromosomes reveals the influence of transcription regulation and chromatin structure on origin selection. *Mol Biol Cell* **21**, 393-404 (2010).

8. Cadoret, J.C. et al. Genome-wide studies highlight indirect links between human replication origins and gene regulation. *Proc Natl Acad Sci U S A* **105**, 15837-42 (2008).

9. Arlt, M.F. & Glover, T.W. Inhibition of topoisomerase I prevents chromosome breakage at common fragile sites. *DNA Repair (Amst)* **9**, 678-89 (2010).

10. Guelen, L. et al. Domain organization of human chromosomes revealed by mapping of nuclear lamina interactions. *Nature* **453**, 948-51 (2008).
